# The absence of protein Y4yS affects negatively the abundance of T3SS *Mesorhizobium loti* secretin, RhcC2, in bacterial membranes

**DOI:** 10.3389/fpls.2015.00012

**Published:** 2015-01-30

**Authors:** Virginia Mercante, Cecilia M. Duarte, Cintia M. Sánchez, Andrés Zalguizuri, Gustavo Caetano-Anollés, Viviana C. Lepek

**Affiliations:** ^1^Instituto de Investigaciones Biotecnológicas “Dr. Rodolfo A. Ugalde,” Universidad Nacional de San MartínBuenos Aires, Argentina; ^2^Evolutionary Bioinformatics Laboratory, Department of Crop Sciences, University of IllinoisUrbana-Champaign, USA

**Keywords:** symbiosis, rhizobium, rhizobia, secretion system, secretin, pilotin

## Abstract

*Mesorhizobium loti* MAFF303099 has a functional type III secretion system (T3SS) that is involved in the determination of nodulation competitiveness on Lotus. The *M. loti* T3SS cluster contains gene *y4yS* (*mlr8765*) that codes for a protein of unknown function (Y4yS). A mutation in the *y4yS* gene favors the *M. loti* symbiotic competitive ability on *Lotus tenuis* cv. Esmeralda and affects negatively the secretion of proteins through T3SS. Here we localize Y4yS in the bacterial membrane using a translational reporter peptide fusion. *In silico* analysis indicated that this protein presents a tetratricopeptide repeat (TPR) domain, a signal peptide and a canonical lipobox LGCC in the N-terminal sequence. These features that are shared with proteins required for the formation of the secretin complex in type IV secretion systems and in the Tad system, together with its localization, suggest that the *y4yS*-encoded protein is required for the formation of the *M*. *loti* T3SS secretin (RhcC2) complex. Remarkably, analysis of RhcC2 in the wild-type and *M. loti y4yS* mutant strains indicated that the absence of Y4yS affects negatively the accumulation of normal levels of RhcC2 in the membrane.

## Introduction

**Figure 1:**
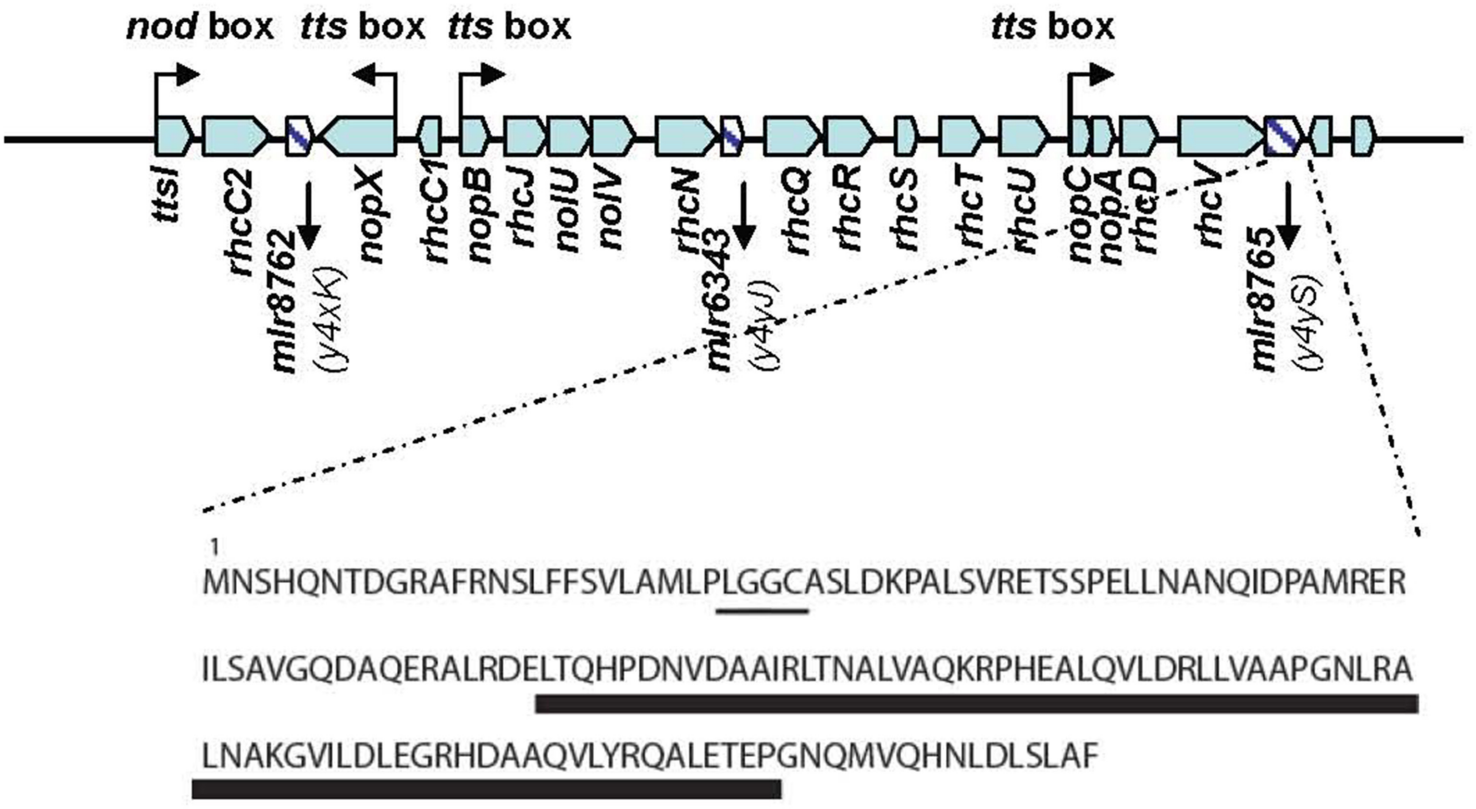
**The T3SS locus of MAFF303099**. Predicted *cis*-elements are shown. Three open reading frames (ORFs) corresponding to genes coding for unknown proteins are shown as hatched bars. Characteristic features of the protein coded by the *y4yS* gene are shown. The lipobox and the region containing the TPR domain are underlined by a thin and a wide line respectively.

Type III secretion systems (T3SSs) are present in several pathogenic bacteria (Viprey et al., 1998; Cornelis, 2002). The T3SS apparatus is a multiprotein complex that delivers effector proteins into the host cell and participates in virulence determination (Galán, 2001; Cornelis, 2002; Alfano and Collmer, 2004). Several of the core protein constituents of the complex are secreted into the bacterial envelope via the universal *sec*-dependent secretion pathway (Francis, 2010). Type III secretion systems also present T3SS-dependent extracellular appendages that link bacteria to their hosts (Saad et al., 2008). In animal pathogens these appendages are called needle structures. When the needle comes into contact with a host cell, synthesis of a translocation pore composed of different bacterial proteins (termed translocators) occurs in the host plasma membrane (Saad et al., 2008). T3SSs are also present in some rhizobia species (Krause et al., 2002; Marie et al., 2004; Sánchez et al., 2009). Flavonoids and NodD induce the expression of rhizobial T3SS components and effectors since the gene encoding the transcriptional factor TtsI contains a *nod* box consensus sequence in its promoter region (Krause et al., 2002; Marie et al., 2004). TtsI binds to *tts* boxes (TB motifs) in the promoter regions of genes encoding T3SS components, inducing their transcription (Wassem et al., 2008). *Mesorhizobium loti* MAFF303099 has a functional T3SS (Sánchez et al., 2009; Okazaki et al., 2010). The T3SS gene cluster is part of the symbiotic island (Kaneko et al., 2000a,b). Regulation of the *M. loti* MAFF303099 T3SS is similar to that of other rhizobia; a *nod* box precedes its *ttsI* gene homolog (Figure 1) (Sánchez et al., 2009). The cluster of T3SS genes of MAFF303099 also contains conserved TB motifs upstream of the orthologs of *nopC* (*mlr8768*), *nopX* (*mll6337*), and *nopB* (*mlr8763*) (Krause et al., 2002) (Figure 1). Several proteins secreted through the rhizobial T3SS have been shown to affect the nodulation process (Bartsev et al., 2004; Skorpil et al., 2005; Rodrigues et al., 2007; Dai et al., 2008; Kambara et al., 2009; Sánchez et al., 2012). Evidence for T3SS effector translocation to the plant host cell cytoplasm has been observed in the case of several proteins (Schechter et al., 2010; Wenzel et al., 2010; Kimbrel et al., 2013). However, translocation during rhizobial nodulation has been observed only for *Sinorhizobium fredii* USDA257 NopP and *Bradyrhizobium japonicum* NopE1/NopE2 (Schechter et al., 2010; Wenzel et al., 2010). Depending on the nodulated legume, a mutation affecting *M. loti* T3SS functionality can alter its nodulation competitiveness (Sánchez et al., 2012). Genes that code for proteins secreted by this system in *M. loti* and with functionality in nodulation competitiveness (*mlr6316*, *mlr6331*, *mlr6361*, and *mlr6358*) were localized in the symbiotic island, outside of the T3SS cluster (Hubber et al., 2004; Sánchez et al., 2009, 2012).

The *M. loti* MAFF303099 T3SS cluster, which contains all the conserved genes required for the formation of the T3SS apparatus, also harbors an additional three genes, *mlr8762*, *mlr6343*, and *mlr8765*, to which no function has yet been demonstrated (Figure 1). *mlr8762* codes for a putative lipoprotein with homology to a protein of *Caulobacter crescentus* involved in the assembly of the extracellular filament (CpaD) (Skerker and Shapiro, 2000; Tampakaki, 2014; Rhizobase data bank). *mlr6343* codes for a protein similar to members of the T3SS SctO protein family with unknown function. *mlr8765* is a homolog to the *y4yS* gene of Rhizobium sp. NGR234, *B. japonicum* USDA110, and *S. fredii* (Marie et al., 2001; Gazi et al., 2012). The *M. loti y4yS* (*mlr8765*) gene belongs to a cluster of open reading frames (ORFs) that present a *tts* box upstream the *nopC* gene (Figure 1). The gene codes for a small unknown protein (165 aa) with a tetratricopeptide repeat (TPR) domain. TPR domains are imperfect 34-amino acid repeats often arranged in tandem arrays (Edqvist et al., 2006) that are involved in protein-protein interactions and the assembly of multiprotein complexes (D'Andrea and Regan, 2003). TPR domains were described in several T3SS proteins such as chaperones, regulators and exceptionally in one T3SS effector. TPR domains are found in class II and class V T3SS chaperones. Class II T3SS chaperones are translocator-chaperones and class V T3SS chaperones are required for T3SS needle formation in pathogens (Sun et al., 2008; Francis, 2010). T3SS of rhizobia have pili instead of a needle (Saad et al., 2008; Abby and Rocha, 2012). NopX, NopA, and NopB have been described as components of rhizobial T3SS pili where NopX has been suggested to be the translocator protein in the system (Marie et al., 2001; Saad et al., 2008). No chaperone for T3SS effectors (named class I chaperones) or for pili components has been described for *M. loti* T3SS until now. The existence of tetratricopeptide-like repeats has also been reported in transcriptional regulators of T3SS such as HilA from *Salmonella enterica* and HrpB from *Ralstonia solanacearum* (Pallen et al., 2003). Also a T3SS effector of Xanthomonas (PthA) was found to have a TPR domain (Murakami et al., 2010). It has also been reported that TPR proteins are involved in the functionality of other secretion systems, including pilotins and some accessory proteins of type IV secretion systems (T4SS) (Korotkov et al., 2011; Koo et al., 2012). Pilotins are small membrane lipoproteins required for the localization and/or stability of the secretin complex formed at the outer membrane (OM) in T2SS, T3SS, and T4SS (Koo et al., 2012). The secretin complex is a homo-multimeric complex that forms a gated channel in the OM, which opens to allow passage of proteins (Koo et al., 2012). Very much as every known OM protein, secretins are synthesized in the cytoplasm as precursors with N-terminal signal sequence, which is essential for translocation across inner membrane by the Sec system (Bos and Tommassen, 2004). Several integral OM proteins are targeted to and insert into this membrane through a cascade of interactions with periplasmic chaperones, with peripheral lipoproteins and with an integral OM lipoprotein complex called the BamA complex (Collin et al., 2011). However, the targeting to the OM of some secretins is independent of the BamA complex and only requires the binding to a specific pilotin (Collin et al., 2011). Pilotins have a type II N-terminal signal sequence followed by a conserved cysteine, which allows the protein to be lipidated and transferred from the inner membrane to the inner leaflet of the OM by the Lol system (Okuda and Tokuda, 2011). Then, some secretins transit to the OM together with pilotin and the corresponding Lol protein (LolA) (Collin et al., 2011). Some secretins are indeed lipoproteins that are directly transported by the Lol system without the requirement of pilotins (Viarre et al., 2009). As was mentioned earlier, pilotins and some accessory proteins were also described as required for secretin monomer and/or secretin complex stability (Koo et al., 2012). Putative TPR domains were also described in an inner membrane accessory protein for type IV pili secretin complex formation FimV (Wehbi et al., 2011). A TPR protein (TadD) appeared to be also required for the formation of the secretin complex in the Tad system of *Aggregatibacter actinomycetemcomitans* (Clock et al., 2008). T3SSs have a secretin complex at the OM and require pilotins for their formation. However, no T3SS pilotin or accessory protein has been described to have TPR domains (Koo et al., 2012).

The aim of the present work was to determine the function of the protein encoded by the *M. loti y4yS* gene.

## Materials and methods

### Bacterial strains and growth media

The bacterial strains and plasmids used in this study are listed in Supplemental Table 1. *Escherichia coli* strains were grown at 37°C in Luria-Bertani media. MAFF303099 strains were grown at 28°C in AB minimal medium (Douglas et al., 1985) supplemented with sucrose (0.5% wt/vol). When necessary, antibiotics were added to the following final concentrations: gentamicin (Gm) at 30 μg/ml, ampicillin (Amp) at 100 μg/ml, neomycin (Nm) at 100 μg/ml, and tetracycline (Tc) at 10 μg/ml for *E. coli* or 1 μg/ml for *M. loti*. For T3SS induction, naringenin was added to cultures at an OD 600 nm of 0.1, to a final concentration of 1 μM.

### Generation of *M. loti y4yS* mutant and complementation

The oligonucleotide primers *mlr8765*UpF, *mlr8765*UpR, *mlr8765*DwF, and *mlr8765*DwR (Supplemental Table 1) were designed to amplify the flanking regions of *mlr8765*. The *Hind*III and *BamH*1 restriction endonuclease sites for the upstream flanking region and *BamH*1 and *Xba*I sites for the downstream flanking region were incorporated into the respective forward and reverse primers. The PCR products were ligated to the pGEMTeasy vector and the appropriate orientation for each was selected, resulting in the generation of pGEMUp*8765* and pGEMDw*8765*. pGEMDw*8765* was digested with *BamH*I plus *Spe*I and the insert was ligated into pGEMUp*8765* digested with the same enzymes. Clones containing pGEMTeasy with the two inserts were selected (pGEMUpDw*8765*). A Gm cassette devoid of the transcriptional terminator sequence (Ugalde et al., 2000) was introduced using the created *BamH*1 site into plasmid pGEMUpDw*8765*, resulting in the generation of pGEMUpDw*8765*::Gm. Gm cassette orientation was selected in the *mlr8765* gene orientation. The gene fragment containing the Gm cassette was cut out of the plasmid with *Hind*III and *Xba*I and ligated with pK18*mob*Tc (Sánchez et al., 2009). The resulting plasmid (pK18mobTc-UpDw*y4yS*::Gm) was used to transform the *E. coli* S17 λ pir strain and then introduced by biparental conjugation into *M. loti* MAFF303099. Gm-resistant clones were isolated and double recombination events were selected by testing sensitivity to Nm and Tc. On this basis, the mutant named *y4yS* strain was selected. By means of PCR, we also confirmed that the mutant generated was the result of a double crossover event.

For mutant complementation, oligonucleotide primers *mlr8765*UpComp and *mlr8765*DwComp (Supplemental Table 1) were designed to amplify the entire *mlr8765* gene. *Hind*III and *BamH*1 restriction endonuclease sites were incorporated into the forward and reverse primers respectively. The amplified fragment was cloned into plasmid pBBR1MCS-4 under the *lac* promoter activity (constitutive in rhizobium), and then introduced by triparental conjugation into the *y4yS* strain.

Plasmid pMP2112 was introduced by triparental conjugation into the MAFF303099 and *y4yS* strains.

### Construction of 3xFlag translational fusions

The *mlr8765* gene was amplified with oligonucleotide primers *mlr8765*FlagUp and *mlr8765*-FlagDw. The *BamH*1 and *Nco*I restriction endonuclease sites were incorporated into the forward and reverse primers respectively. pBAD-*y4yS-*1 was constructed by cloning the amplified gene sequence into vector pBAD 3x FLAG (Supplemental Table 1). The amplified fragment was sequenced to eliminate any possible alteration. The fragment containing the fusion to the 3x FLAG sequence was cut with restriction enzymes *BamH*1 and *Hind*III, and then cloned into pBBR1MCS-4 in the orientation of the *lac* promoter. The resulting plasmid (Supplemental Table 1) was transferred to *y4yS* pMP2112 by triparental mating.

Oligonucleotide primers *mlr8765*Up and *mlr8765*-FlagDw were used to integrate the *y4yS*-FLAG fusion into the chromosome. The amplified fragment in this case did not contain the N-terminal gene sequence. This eliminates the expression of *y4yS* that has not been fused to Flag. pBAD-*y4yS*-2 was constructed by cloning the amplified gene sequence into the vector pBAD 3x FLAG (Supplemental Table 1). The amplified fragment was sequenced to eliminate any possible alteration. The fragment containing the fusion to the 3x FLAG sequence was cut with restriction enzymes *BamH*1 and *Hind*III, and then cloned into pK18mobTc (Sánchez et al., 2009). The plasmid was introduced by biparental mating into the MAFF303099 pMP2112 strain. The single homologous recombination event was selected by searching Tc resistant strains and confirmed by PCR using oligonucleotides complementary to the vector sequence.

For chromosomal integration of the *mlr6335*-FLAG fusion, the C-terminal fragment of the gene was amplified with oligonucleotide primers *mlr6335*-FlagUp and *mlr6335*-FlagDw. The same procedure described above for integration of the *mlr8765* fusion was carried out. The only difference was that the fragment containing the fusion to the 3xFlag sequence was cloned into pK18mob (Schäfer et al., 1994) and then a Tc cassette was introduced into the *Hind*III restriction site in the new plasmid. This allowed the expression of the gene downstream *mlr6335* (*mlr8762* gene) under the *lac* promoter activity of pK18mob. The resulting plasmid was introduced by biparental mating both into the MAFF303099 pMP2112 and *y4yS* pMP2112 strains. The single homologous recombination event was selected as described above.

### Cell fractionation

Bacterial protein extraction involved centrifuging 1 ml of the bacterial cultures and resuspending the resulting pellets in SDS-PAGE sample buffer (50 mM Tris-HCl pH 6.8, 2% SDS, 0.1% Bromophenol Blue, and 10% glycerol) with the addition of 2% β-MSH and 0.8 M Urea. Bacterial membranes were isolated by cellular lysis using osmotic shock. Briefly, cells were harvested by centrifugation. Bacterial pellet was resuspended in lysis buffer (50 mM Tris-HCl pH 8.0, 5 mM EDTA, 12% Sucrose, 2 mM of phenylmethylsulfonyl fluoride, 0.02 mg/ml of lisozyme, and protease inhibitor cocktail from Sigma). After overnight incubation at 4°C, eight volumes of cold water were vigorously added to the suspension. After the addition of 10 μg/ml of DNAse and MgCl_2_ (final concentration 10 mM), the suspension was incubated 1 h at 4°C and then centrifugated at 3000 × g. Supernatant was subjected to ultracentrifugation by 4 h at 164,000 × g. The soluble fraction containing cytoplasmic and, presumably, periplasmic proteins, was precipitated with 10% of TCA. The pellet comprising bacterial membranes was resuspended in 3% ZW-3-14 with 250 mM NaCl to increase membrane proteins solubilization (Guilvout et al., 2006) and incubated at room temperature for 1 h. Then phenol treatment was made to dissociate possible multimers as was previously described (Guilvout et al., 2006). Outer and inner membranes were fractionated by differential detergent solubilization of total membranes as previously described (Koster et al., 1997) using 2% Sarkosyl. The Sarkosyl-soluble fraction contained the inner membrane proteins, whereas the pellet contained the OM proteins. Pellets were resuspended in SDS-PAGE sample buffer and heated at 65°C or at 100°C for 5 min. In the latter, β-MSH and Urea were added to the cracking buffer.

Inner and outer membrane protein-containing fractions were separated also by equilibrium density gradient centrifugation according Osborn et al. (1972). Fraction aliquots were analyzed to determine protein content (Bio-Rad protein assay) and NADH oxidase activity (as described by Osborn et al., 1972). For immunoblot analysis, equivalent volumes of each fraction were precipitated with 10% TCA and heated in SDS-PAGE sample buffer at 100°C.

### Isolation of extracellular proteins

Supernatant protein extractions were carried out by direct trichloroacetic acid precipitation as previously described (Sánchez et al., 2009).

### Analysis of proteins by gel electrophoresis

Proteins were separated using sodium dodecyl sulfate polyacrylamide gel electrophoresis (SDS-PAGE) and then stained using silver nitrate. For immunoblotting, the anti-NGR234 strain NopA, the anti-NGR234 strain NopX (Marie et al., 2004), the anti-Brucella Omp19 or a commercially available anti-FLAG M2 monoclonal antibody (Sigma) were used. SuperSignal West Femto reagent (Thermo Scientific) was used as a substrate for horseradish peroxidase to detect the proteins encoded by the chromosome-integrated translational fusions (Y4yS-3xFLAG and RhcC2-3xFLAG). When indicated, detection of mouse anti-FLAG and rabbit anti-Omp19 antibodies was made with fluorescent antibodies anti-mouse and anti-rabbit and subsequent revealing analysis in the Li-Cor, Odyssey equipment.

### Competitive analysis

For competitive analysis, the indicated strains were mixed together in equal amount and used to inoculate Lotus plants as previously described (D'Antuono et al., 2005). The proportion of nodules occupied by each strain was determined as previously described (Sánchez et al., 2009). The strain that occupies the higher proportion of nodules is the strain that presents higher competitiveness. Statistical analyses were carried out by ANOVA and the Chi-square test.

### Bioinformatic analysis

The amino acid sequences of TPR proteins were aligned using MUSCLE v(3.8.31) (Edgar, 2004). Phylogenetic trees were recovered using the maximum likelihood optimality criterion and the JTT matrix-based model (Jones et al., 1992). A bootstrap consensus tree inferred from 1000 replicates was taken to represent the evolutionary history of the taxa analyzed (Felsenstein, 1985). Branches corresponding to partitions reproduced in less than 45% bootstrap replicates were collapsed. The percentages of replicate trees in which the associated taxa clustered together in the bootstrap test (1000 replicates) was shown next to the branches (Felsenstein, 1985). Initial trees for the heuristic search were obtained automatically by applying the Neighbor-Joining and BioNJ algorithms to a matrix of pairwise distances estimated using a JTT model, and then selecting the topology with superior log likelihood value. All positions containing gaps and missing data were eliminated. The final dataset had a total of 106 positions. Evolutionary analyses were conducted in MEGA6 (Tamura et al., 2013).

The mirror tree online server (Ochoa and Pazos, 2010) was used to assess the co-evolutionary relationship between *M.loti* Mlr8765 and Mlr6335. Two homologous groups were created for each reference protein, the first one containing high scoring BLAST hits retrieved from NCBI NR database with a coverage >60% (see Supplementary text 1 and Supplementary text 2), and the second one, containing the first group plus seven distant elements of TPR secretins/pilotins pairs that are known to interact as a part of the secretion system (see Supplementary text 3 and Supplementary text 4). The four groups were aligned using ClustalX v2.1, with the standard settings and submitted to the web server where phylogenetic trees are obtained from these alignments with the neighbor-joining (NJ) algorithm implemented in ClustalW (Chenna et al., 2003) using bootstrap (100 repeats) and excluding gaps for the calculation. The distance matrices are obtained by summing the branch lengths that separate each pair of proteins in the tree. Instead of calculating the complete matrices the tree similarity between the two families is calculated as the correlation between their distance matrices according to the standard equation (Pazos and Valencia, 2001):

$$r=\frac{\sum_{i=1}^{n}\left(R_{i}-\bar{R}\right)\left(S_{i}-\bar{S}\right)}{\sqrt{\sum_{i=1}^{n}{\left(R_{i}-\bar{R}\right)}^{2}}\sqrt{\sum_{i=1}^{n}{\left(S_{i}-\bar{S}\right)}^{2}}}$$

Where *n* is the number of elements of the matrices, that is, *n* = (*N*^2^-*N*)/2 being *N* the number of common organisms, *R_i_* are the elements of the first distance matrix (the distance among all the proteins in the first multiple sequence alignment), *S_i_* is the corresponding value for the second matrix and *R* and *S*, are the averages of *R_i_* and *S_i_* respectively.

## Results and discussion

### The *M. loti y4yS* mutant strain exhibits the same nodulation phenotype as the T3SS mutant strain rhcN

**Figure 2:**
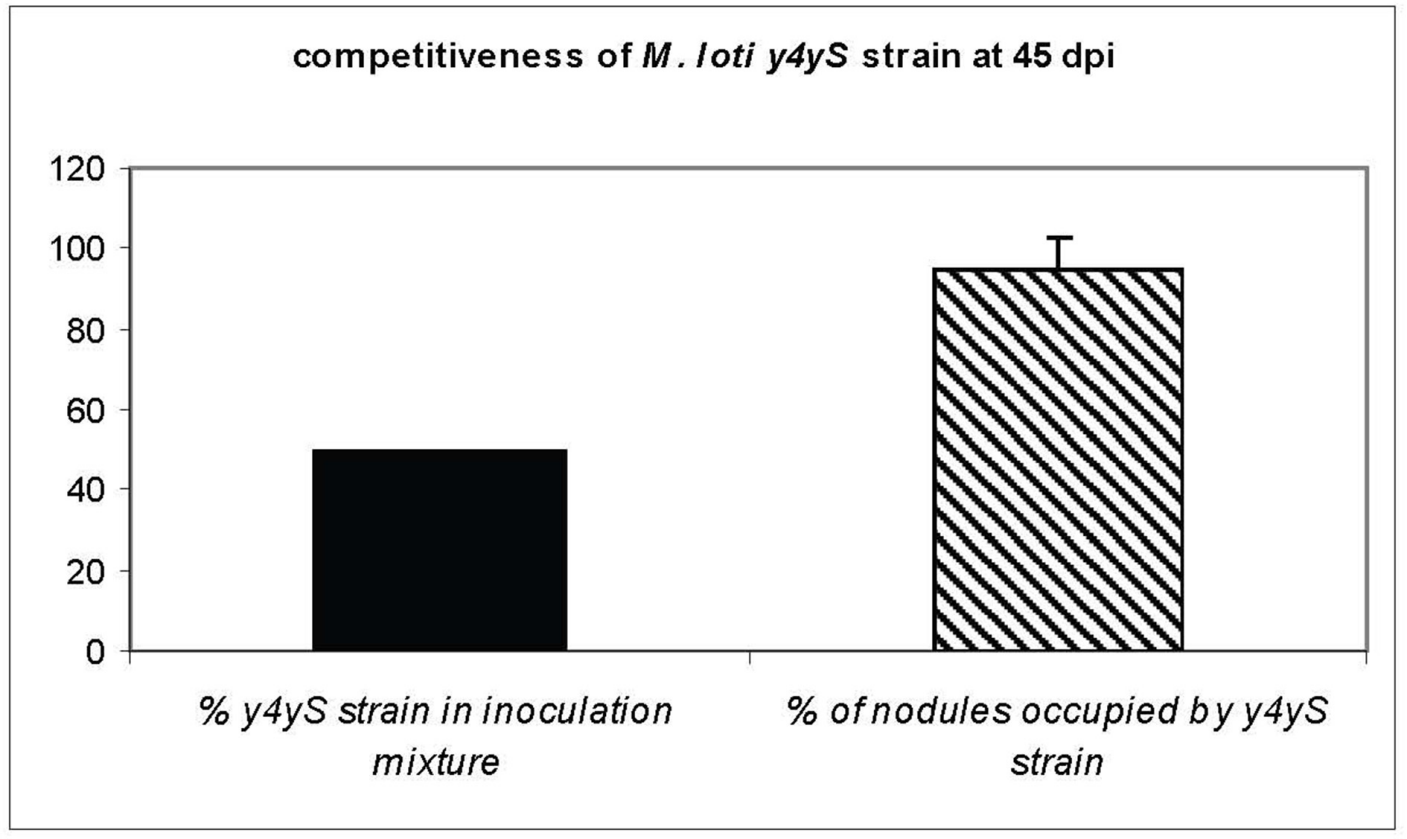
**Competition assay on *Lotus tenuis* cv. Esmeralda**. Plants were co-inoculated with an equal mixture of the wild-type and *y4yS* mutant strains. The percentage of nodules occupied by the *y4yS* strain at 45 days post-inoculation (dpi) is shown.

A MAFF303099 *y4yS* mutant was generated by the integration of a non-polar Gm-resistance cassette into the gene. Previously, we described that the *M. loti rhcN* mutant strain is more competitive than the wild-type strain in co-inoculation assays on *Lotus tenuis* cv. Esmeralda (Sánchez et al., 2012). *M. loti* RhcN protein shows homology to T3SS ATPases and is required for *M. loti* T3SS functionality (Sánchez et al., 2009). On the other hand, mutants in one, two or three of the genes coding for the putative effectors secreted by this system showed decreased competitiveness than the wild-type strain (Sánchez et al., 2009). Taking into account that this assay allows us to infer the effect of T3SS mutation on nodulation phenotype, we compared the competitiveness of the *y4yS* mutant strain with that of the wild-type strain on *L. tenuis* cv. Esmeralda. At 45 days post inoculation (dpi) with a rhizobial mixture (1:1) of the wild-type and the *y4yS* mutant strains, 95.5% of the nodules were occupied only by the mutant strain and the remaining 4.5% only by the wild-type strain. No mixed nodules were observed (Figure 2). The wild-type strain inoculated alone showed a normal nodulation phenotype (data not shown). The fact that the nodulation phenotype of the *y4yS* mutant on *Lotus tenuis* cv. Esmeralda resembles the phenotype observed when the mutation affects system functionality suggests that the protein codified by *y4yS* (hereafter Y4yS) is also required for *M. loti* T3SS functionality. Analysis of the *in vitro* mutant growth rate showed no differences with the wild-type strain (data not shown).

### Type III secretion is abolished in the *y4yS* mutant strain

We have previously demonstrated *M. loti* T3SS functionality by analyzing protein secretion to the culture supernatant (Sánchez et al., 2009). Among the proteins secreted by rhizobial T3SS are pili components such as NopX and NopA (Saad et al., 2008; Sánchez et al., 2009). It has been described that while a mutation in NopX does not affect NopA secretion in *Rhizobium* sp. strain NGR234, a mutation in NopA abolishes NopX secretion (Deakin et al., 2005). To discriminate between Y4yS being the NopX chaperone (putative class II chaperone) or the NopA chaperone (putative class V chaperone case), we decided to determine the effect of a mutation in *y4yS* on the secretion of the proteins. As was previously described (Sánchez et al., 2009, 2012), all the strains used (here and thereafter) contain the plasmid pMP2112 (López-Lara et al., 1995), which constitutively expresses *nodD* of *Rhizobium leguminosarum* and allows the *in vitro* induction of *M. loti* T3SS with naringenin.

**Figure 3:**
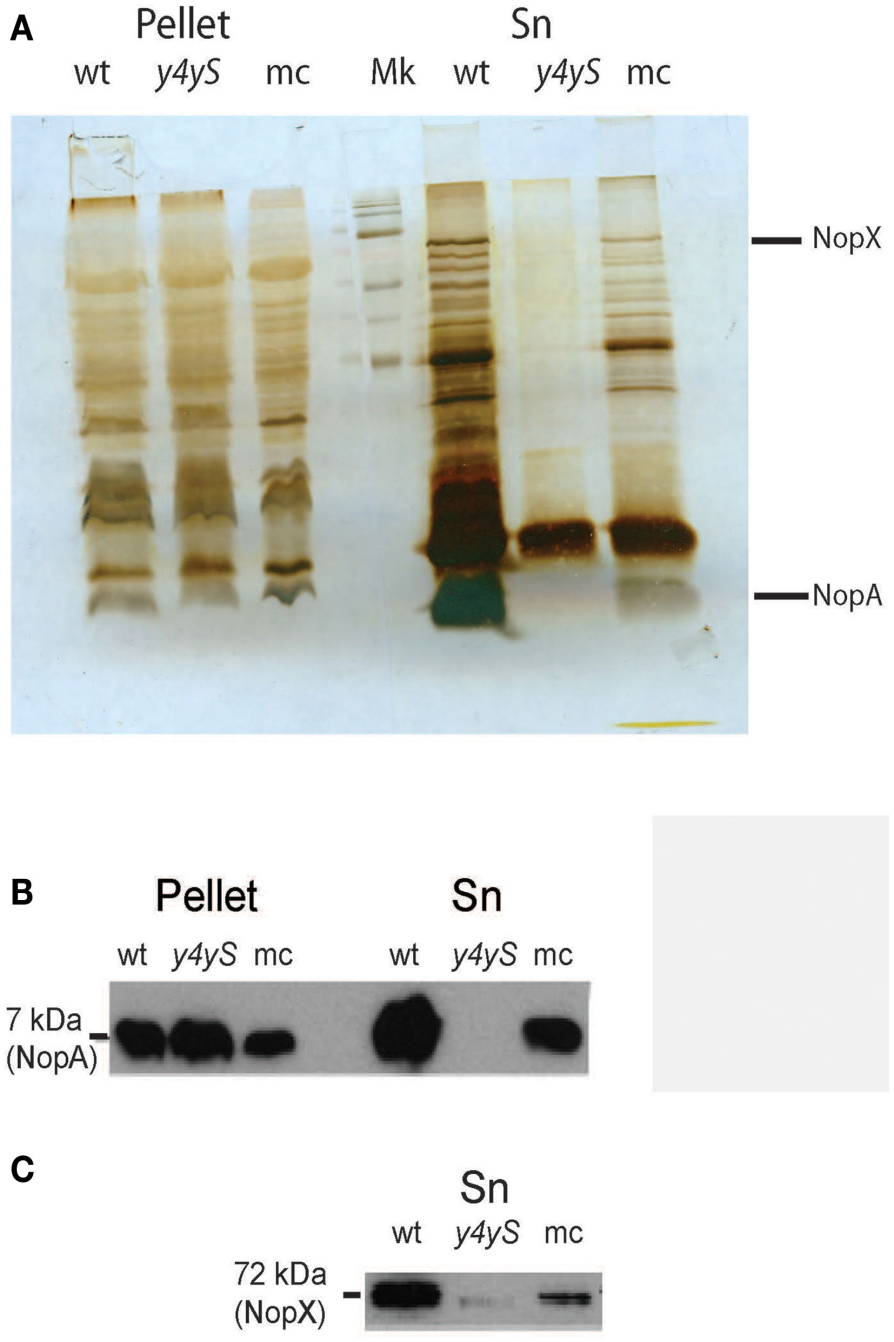
**Analysis of T3SS secreted proteins in the wild-type, *y4yS* mutant, and *y4yS* mutant complemented strains**. Supernatant (Sn) and intracellular proteins (pellet) were isolated from MAFF303099 (wt), its *y4yS* mutant (*y4yS*), and the mutant complemented with a plasmid of moderate copy number containing the full-length *y4yS* gene under the *lac* promoter (mc). Bacteria were grown in T3SS inducing conditions. All bacteria contain plasmid pMP2112. Proteins were separated by 15% SDS-PAGE, stained with silver nitrate **(A)** or transferred to membranes and probed with anti-NopA antibody **(B)** or anti-NopX antibody **(C)**.

The silver stained gel showed that protein secretion to the culture supernatant in the T3SS inducing conditions was negatively affected in the *y4yS* mutant strain (Figure 3A). A Western blot analysis using anti-NopX and anti-NopA confirmed that the secretion of both NopX and NopA, proteins normally secreted by T3SS, was inhibited in the mutant strain (Figures 3B,C). The secretion defect was reversed by mutant complementation with a gene copy into a plasmid of moderate copy number (Figure 3). Therefore, we conclude from this experiment that Y4yS is not a NopX chaperone because not only NopX secretion was inhibited but also NopA secretion was negatively affected. We cannot exclude that Y4yS is the NopA chaperone.

Very much as in the wild-type, NopA was detected in the mutant pellet. This indicates that the defect in protein secretion was not a consequence of a defect in NopA expression and discards a negative T3SS transcriptional regulator role for the protein coded by *mlr8765*. NopX could not be detected in wild-type or mutant pellets (data not shown). T3SS secreted proteins of some rhizobia could be detected in the culture supernatant but not in the bacterial pellet even in the case of mutants that are affected in secretion (Deakin et al., 2005; Krishnan et al., 2007). It was speculated that the accumulation of Nops inside the cell could be deleterious to the rhizobial cells and thus subjected to rapid degradation. This could be also true for NopX in *M. loti*.

### Y4yS is localized in bacterial membranes

**Figure 4:**
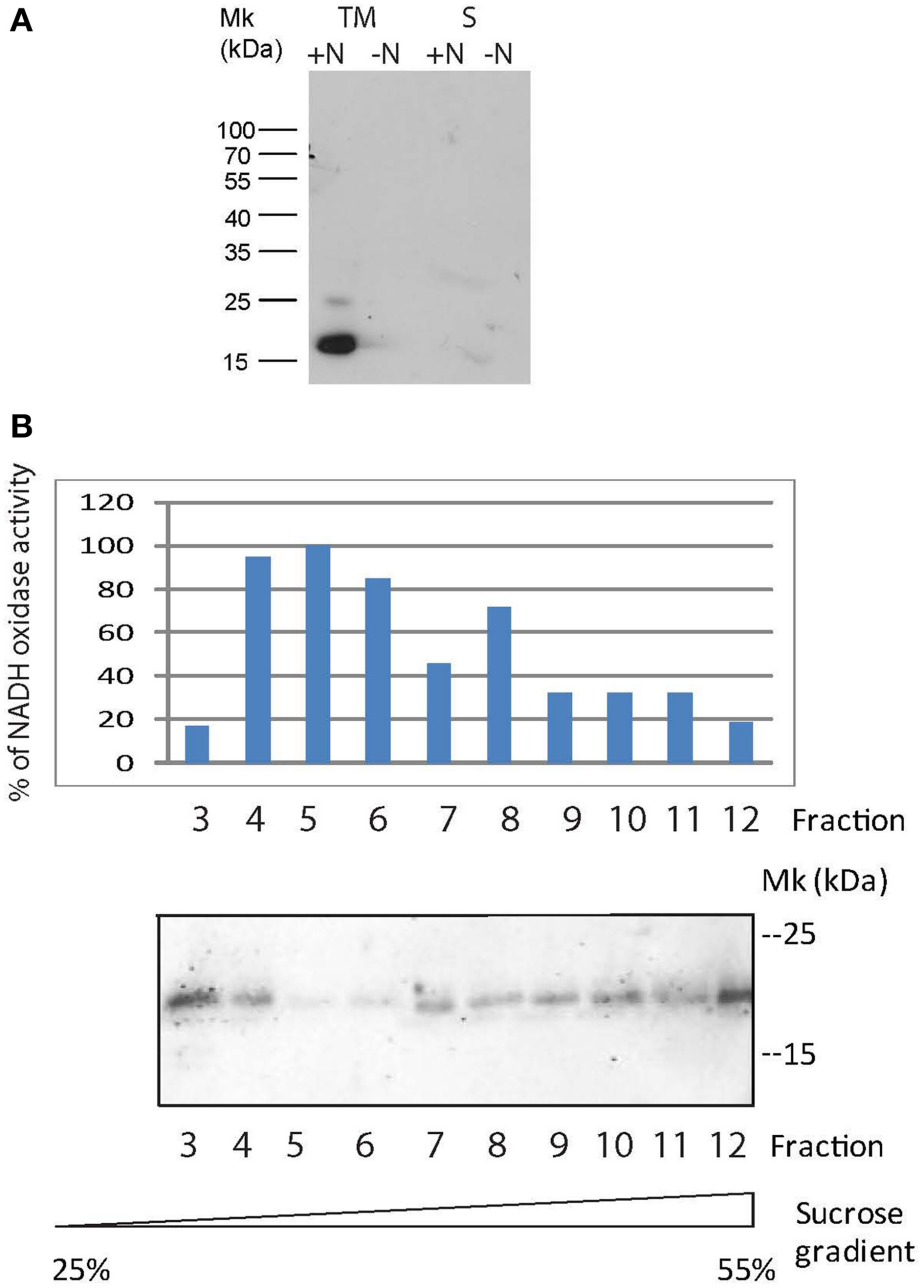
**Expression and localization of the 3x FLAG Y4yS fused protein. (A)** Total membranes (TM) and fractions corresponding to the cytoplasm and periplasm (S) of the MAFF303099 strain with sequence encoding the 3xFLAG Y4yS fused protein integrated into the bacterial chromosome. The two bacteria contain plasmid pMP2112. Proteins were separated by 10 % SDS-PAGE and then immuno-blotted and probed with an anti-FLAG antibody. Positions of size markers loaded onto the gels are labeled (in kDa). ±N indicate bacterial culture in the presence or absence of naringenin. **(B)** Subcellular localization of Y4yS-FLAG expressed from a pBBR1MCS-4 plasmid into an *y4yS* mutant strain was determined by sucrose density gradient centrifugation. Inner and outer membranes were fractionated as described in Materials and Methods. Fractions were collected as 1-ml aliquots from the top of a discontinuous sucrose gradient. Fractions enriched in the inner membranes were identified by monitoring NADH oxidase activity. Enzyme activity was expressed as percentage of maximal activity. Y4yS-FLAG was detected by immunobloting with anti-FLAG (from mouse) and fluorescent anti-mouse antibodies. Bacteria contain plasmid pMP2112.

We determined the cellular localization of Y4yS. Previously, the protein fused to the triple (3x) copy of the FLAG peptide was introduced into the *M. loti y4yS* mutant strain, cloned into a plasmid of moderate copy number under a constitutively active promoter in rhizobia. A Western blot analysis of total bacterial extract showed the presence of a band between 15 and 25 kDa, in agreement to the theoretical molecular weight of the protein (16 kDa) (Supplementary Figure 1). Localization analysis detected the fused protein both in the membrane and cytoplasm fractions although higher levels were detected in membranes (data not shown). The possibility of over expression artifacts led us to integrate the tagged protein into the *M. loti* genome via a single recombination event in order to have only one copy of the fused construct in the cell. Once chromosome integrated, the fused proteins were expressed from the corresponding chromosomal promoter. Western blot analysis of the various cellular fractions showed that Y4yS was localized exclusively in bacterial membranes (Figure 4A).

Western blot results also indicate that Y4yS expression occurred in naringenin-induced culture, which is the condition of induction of *M. loti* T3SS expression (Sánchez et al., 2009) (Figure 4A). This confirms that the *y4yS* gene forms part of an operon of co-transcribed ORFs *nopC*-*nopA*-*rhcD-rhcV*-*y4yS* under a promoter region with a *tts* box localized upstream *nopC* (Tampakaki, 2014).

We then determined the inner or outer membrane localization of the Y4yS-FLAG protein. Attempts to detect the chromosome-encoded fusion protein were unsuccessful probably because of the low protein levels in the cell. We decided to make this determination with bacteria expressing the fused protein from the pBBR1MCS-4 plasmid. Inner and outer membranes were separated by density gradient centrifugation. Results showed that the Y4yS protein is localized both in outer and inner membranes (Figure 4B). Attempts to detect the Omp19 protein by Western blot (an OM porin used as OM marker) were unsuccessful probably because of sample dilution.

### Y4yS presents sequence features of T4SS pilotins and TadD protein

**Figure 5:**
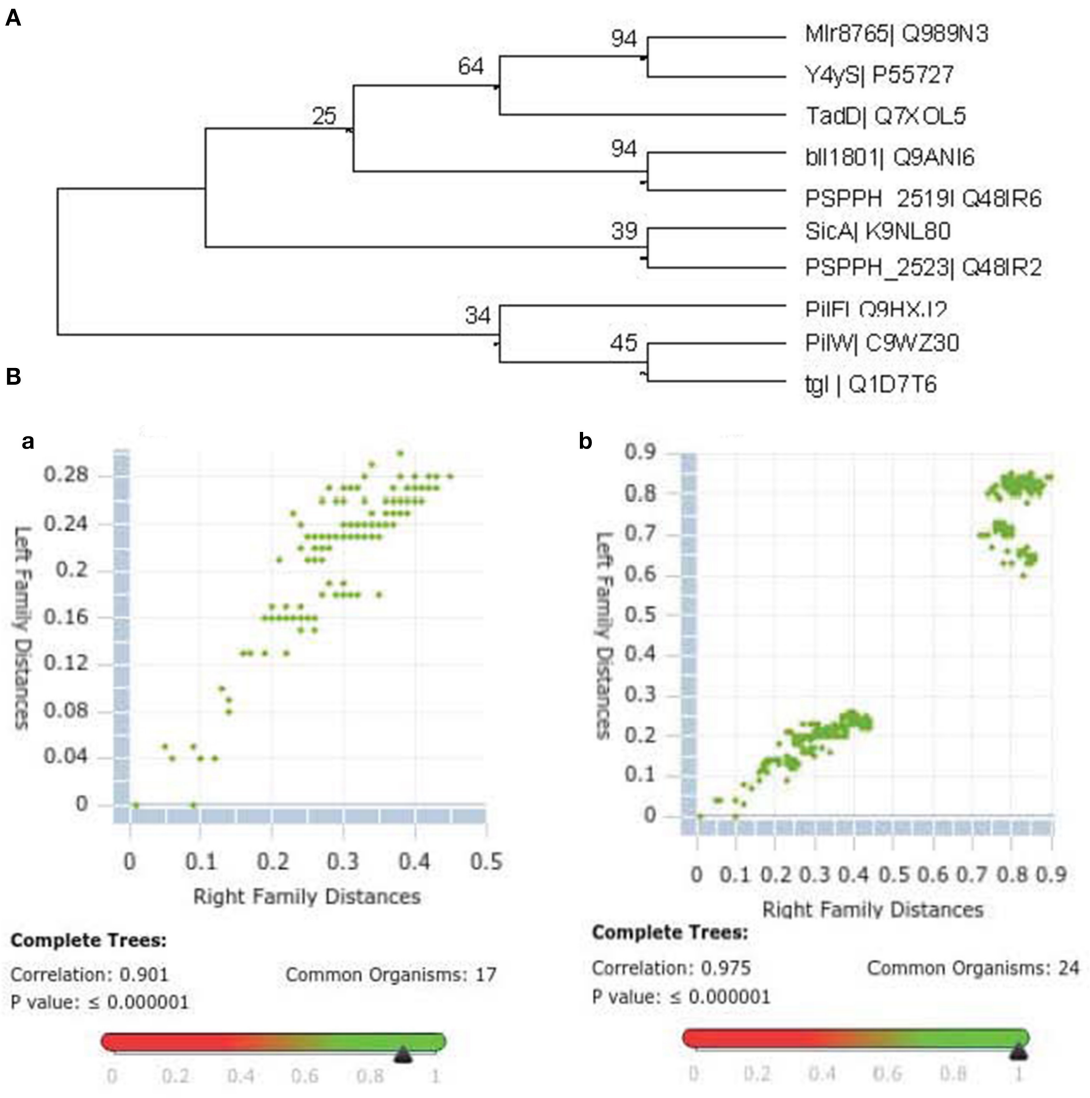
**(A)** Phylogenetic analysis of bacterial TPR proteins involved in secretion systems. Bacterial TPR proteins described in the text were subjected to a phylogenetic analysis as indicated in Materials and Methods. A cladogram could be constructed only with the proteins indicated next to each branch arm. The percentages of replicate trees in which the associated taxa clustered together in the bootstrap test are shown next to the branches. **(B)** Inter-protein distances of two phylogenetic trees represented in a scatterplot showing the conserved correlation between the Left Family Distances corresponding to Mlr8765 homologs and the Right Family Distances corresponding to Mlr6335 homologs. (a) Smaller homolog protein set composed by high identity blast hits. (b) Larger homolog protein set that includes TPR secretins and their respective pilotins or docking proteins to the set of high identity blast hits.

Since T3SS chaperones are generally cytoplasmic proteins, Y4yS membrane localization argues against a chaperone role for this protein (Francis, 2010). However, this role cannot be completely excluded. Two class V chaperones for needle components in *Escherichia coli*, EscG and EscE, were described to be in the inner membrane (Sal-Man et al., 2013). Nevertheless, these proteins do not present TPR domains. TPR proteins have been described in T4SS of *Pseudomonas* (PilF), *Yersinia* (PilF) and *Neisseria* (PilW) and in the Tad system of *A. actinomycetemcomitans* (TadD), where they function as pilotins and docking proteins required for the formation of the secretin complex at the OM (Clock et al., 2008; Koo et al., 2012). These four TPR proteins are localized in bacterial membranes and, in addition to the TPR domain, they present a lipidation site at their N-terminus characterized by a specific consensus motif, the lipobox (V/L)XXC. This motif is characteristic of the processing site of lipoproteins (Wu and Tokunaga, 1986). *In silico* analysis of Y4yS indicates that the protein contains a predicted site for N-terminal cleavage by peptidase II in accordance with its membrane localization and that it presents the characteristic sequence for lipidation LGCC (Figure 1). δ-Blast homology searches to the Y4yS amino acid sequence showed homology, although poor, to PilW (*E*-value 5 × 10^−4^), PilF (*E*-value 4 × 10^−4^), and Aggregatibacter TadD (*E*-value 2 × 10^−4^). Since homology could be due to the presence of the TPR domain in these or other proteins, we decided to apply phylogenetic analysis to several bacterial TPR proteins that were described to be involved in secretion systems. This included TadD from *A*. *actinomycetemcomitans* (Clock et al., 2008), pilotins such as *Pseudomonas* PilF, *Neisseria* PilW, and *Myxococcus* tgl (Koo et al., 2012), T3SS chaperones with TPR domains such as LcrH, PcrH, IpgC, YscG, PscG, and SicA (Francis, 2010; Cerveny et al., 2013), and other unknown TPR proteins that are part of the secretion system gene cluster, the *B. japonicum bll1801* gene and the *Pseudomonas* PSPPH2519 and PSPPH2523 genes (Gazi et al., 2012). The analysis also included the Mlr8765 ortholog in *S. fredii*. The protein encoded by *M. loti mlr8765* was phylogenetically related to few of these proteins, and among them, the most closely related was the TadD protein from *A*. *actinomycetemcomitans* (Figure 5A). The protein alignment is shown in Supplementary Figure 2. It has been recently described that two genes code for proteins with homology to secretins in the Rhizobiales-T3SS family (also referred as Rhc-T3SS), which includes rhizobia and some strains of *Pseudomonas syringae* T3SS (Abby and Rocha, 2012). In *M. loti* MAFF3030999, the two genes are *mlr6335* and *mlr6338*. *mlr6335* codes for RhcC2, which shows homology with the secretins of the Tad (tight adherence) macromolecular transport system present in bacteria such as *Caulobacte*r and *Aggregatibacter* (CpaC and RcpA respectively) (Abby and Rocha, 2012). *mlr6338* codes for a protein that presents homology with the N-terminal part of T3SS secretins (Clock et al., 2008; Abby and Rocha, 2012). A phylogenetic analysis of the T3SS secretins together with secretins from T2SS, type IV pilus, Tad system and filamentous phages showed that rhizobial secretin RhcC2 groups together with secretins from the Tad loci (RcpA) (Abby and Rocha, 2012; Clock et al., 2008). The same study concludes that rhizobia originally had a non-flagellar-T3SS-like secretin, RhcC1, and secondarily acquired the secretin RhcC2 from a Tad locus through a partial homologous gene replacement (Abby and Rocha, 2012). In Aggregatibacter the *tadD* gene is downstream the *tadC* gene. *M. loti* has a cluster of Tad gene homologs (*mlr5593* to *mlr5604*). *M. loti* Tad secretin coded by *mlr5597* gene presents a 32% homology with *M. loti* T3SS secretin RhcC2. A gene localized downstream the *M. loti tadC* gene (*mlr5604*) and in opposite direction to the Tad cluster (*mll5605*) encodes an unknown protein with 32% homology with *M. loti* Y4yS. The above-described data raised the possibility that Y4yS, which shares sequence features and is evolutionarily related to TadD, might be a protein required for the complex formation of RhcC2 (evolutionarily related to the Tad secretin) in *M. loti* MAFF303099 T3SS. The Rhc-T3SS family is subdivided into three subgroups according to the organization of the T3SS core genes (Gazi et al., 2012). T3SS core genes of subgroup I, represented by Rhizobium sp. NGR234, *B. japonicum* USDA 110, *S. fredii*, and *M. loti* MAFF303099, are organized in three segments (Gazi et al., 2012; Tampakaki, 2014). The second fragment in members of this subgroup harbors the genes *rhcD*, *rhcV* and *y4yS*. Since members of Subgroup I have both RhcC2 and Y4yS, our hypothesis could be extended to the four above-mentioned strains. To analyze the existence of an evolutionary relationship between Y4yS to RhcC2, despite being coded in separate segments of the Rhc-T3SS cluster, a comparison between the phylogenetic trees of Y4yS homologs and the respective secretin RhcC2 homologs was made using the Mirror tree online server (Ochoa and Pazos, 2010). Two homologous groups were created for each reference protein as was described in Materials and Methods. Mlr8765 and Mlr6335 showed a high correlation coefficient in both pairs of homologous protein groups with a *P*-value <1e-6 (Figure 5B). Interestingly, the larger group, which included distant homologs, presented a higher correlation score that the smaller and more closed related group. This result suggests that the TPR secretin/pilotin or docking protein and the rhizobial T3SS secretin/Y4yS homolog proteins are coevolving and argues in favor of the existence of a physical interaction between RhcC2 and Y4yS proteins.

### The *M. loti y4yS* mutant strain presents lower RhcC2 protein levels in the bacterial membranes

**Figure 6:**
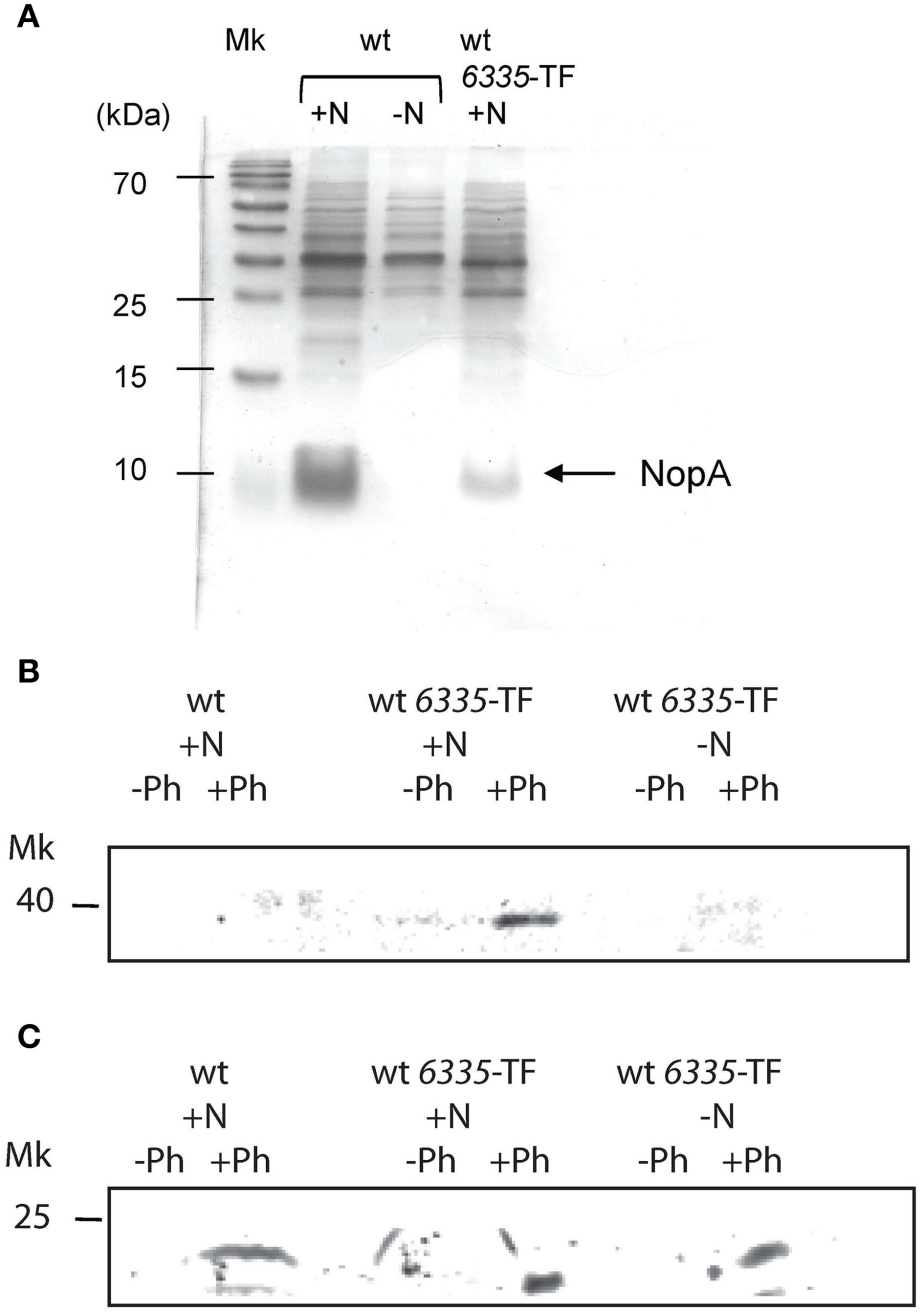
**Detection of the 3xFLAG RhcC2 fused protein in total membrane fraction. (A)** Silver staining of supernatant proteins of MAFF303099 (wt) and MAFF303099 with sequence encoding the 3xFLAG RhcC2 fused protein integrated into the bacterial chromosome (wt *6335*-TF). Total membranes of wt and wt *6335*-TF strains were separated by 12.5% SDS-PAGE and immune-bloted and probed with anti-FLAG antibody (from mouse) **(B)**, and with anti-Omp19 antibody (from rabbit) **(C)**. For detection fluorescent anti-mouse and anti-rabbit antibodies were used. All bacteria contain plasmid pMP2112. ± N: with or without induction with naringenin, ± Ph: with or without phenol treatment.

**Figure 7:**
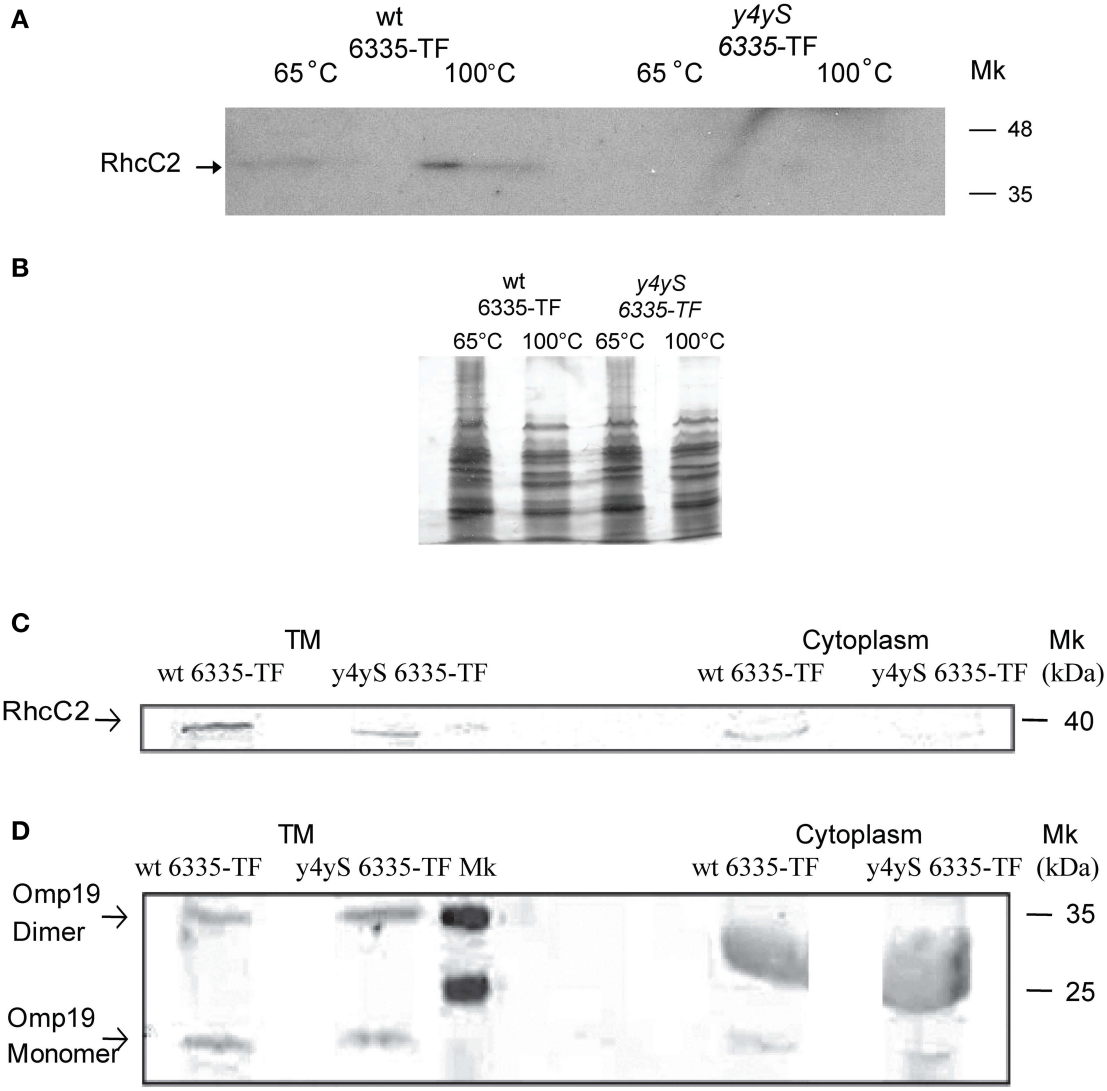
**Detection of the 3x FLAG RhcC2 fused protein. (A)** OMs of wt *6335*-TF and *y4yS* mutant strain with sequence encoding the 3xFLAG RhcC2 fused protein integrated into the bacterial chromosome (*y4yS 6335*-TF) heated at 65°C or 100°C were separated by 15% SDS-PAGE and then immuno-bloted and probed with an anti-FLAG antibody, **(B)** Silver staining of samples described in A separated by 7.5% SDS-PAGE. Total membranes (TM) and cytoplasmic fractions of wt *6335*-TF and *y4yS 6335*-TF strains heated at 100°C, separated by 12.5% SDS-PAGE and then immuno-bloted and probed with anti-FLAG **(C)** and anti-Omp19 **(D)** antibodies and revealed with fluorescent antibodies. All bacteria contain plasmid pMP2112 and all bacterial cultures were made in the presence of naringenin. Positions of RhcC2, and of monomer and dimer of Omp19 are indicated. Positions of size markers loaded onto the gels are labeled (in kDa). Anti-Omp19 antibodies nonspecifically probe a great band both in wt and mutant cytoplasmic fractions between markers of 25 and 35 kDa.

To analyze the involvement of Y4yS in the formation of the *M. loti* RhcC2 complex, we integrated the *mlr6335* gene that codes for RhcC2 fused to the triple (3x) copy of the FLAG peptide into the chromosome of the *M. loti* MAFF303099 and *y4yS* mutant strains. Sequences coding for Flag-tagged proteins were integrated into the chromosome by a single homologous recombination event. Following chromosomal integration, the fused proteins were expressed from the corresponding chromosomal promoter. NopA secretion in inducing conditions was analyzed for the wild-type strain with the integrated fused protein to rule out the possibility of abolishing type III secretion due to the C-terminal modification of RhcC2 or by defects in the gene expressed downstream, *mlr8762*. Figure 6A shows that the isolated strain, although in lower levels than the wild-type strain, still secretes NopA. Western blot analysis using anti-FLAG antibody indicates that a protein of about 35–40 kDa (40 kDa was the expected molecular weight) was detected only in the total membrane fraction of the wild-type strain with the tagged RhcC2 induced with naringenin but not in the total membrane fraction of the wild type strain without tagging nor in the wild type strain with the tagged protein but without induction with naringenin (Figure 6B). The detection of RhcC2 in the total membrane fraction required phenol treatment. Results confirm that RhcC2 protein is expressed under naringenin induction. Consequently, its expression depends on the activity of the upstream promoter with the *tts* box. Detection using anti-Omp19 antibodies shows similar protein levels in the three preparations (Figure 6C). As the secretin complex has been described to have OM localization, we isolated the OM of the wild-type and mutant strain with the tagged RhcC2 protein. Since the secretin complex of the Tad system is resistant to detergent at 65°C but sensitive to boiling (Clock et al., 2008), we analyzed the presence of the RhcC2-3x-FLAG protein in OM of the wild-type and *y4yS* mutant strains by SDS-PAGE electrophoresis after resuspending and heating the samples at 65°C and 100°C in the SDS-PAGE sample buffer. The anti-FLAG antibody detected a monomer of about 40 kDa only in the wild-type OM (Figure 7A). Under silver staining the samples revealed a similar pattern and amount of proteins (Figure 7B). A slight increase in 40 kDa protein levels was observed when the sample was boiled in the SDS-PAGE sample buffer at 100°C instead of at 65°C (Figure 7A). Unfortunately, all attempts to detect the high molecular polymers corresponding to secretin oligomers were unsuccessful. In some systems, it is difficult to observe the secretin complex with Western blots due to problems of complex solubility and efficient transfer of high-molecular-weight species to nitrocellulose (Burghout et al., 2004; Clock et al., 2008). The absence of RhcC2 in mutant OM indicates that the mutation in Y4yS affects the production, stability or localization of RhcC2. The T3SS protein expression is not affected in the *y4yS* mutant so a deficiency in transcription is quite unlikely. In some T3SS systems, secretin is localized in the inner membrane in the absence of pilotin (Koster et al., 1997; Koo et al., 2008), whereas in the Tad system, no endogenous secretin is localized in the whole cell extract in the absence of pilotin and in physiological conditions (Clock et al., 2008). To address this problem we determined total RhcC2 protein levels in the cell. Figures 7C,D show the Western blot results on total membrane and cytoplasmic fractions, of wild type and *y4yS* mutant strains containing the tagged RhcC2 protein. Results indicate that the flagged RhcC2 protein is localized in membranes and that the *y4yS* mutant exhibits lower levels of this protein in total bacterial extract (membranes and cytoplasm), resembling the results observed in the Tad system (Clock et al., 2008). Omp19, the OM marker, can be detected as a monomer and/or a dimer (unpublished results). Figure 7D shows that total Omp19 taken as the sum of monomer and dimer is the same in the two samples that were compared.

## Conclusion

In the present study we determined that a *M. loti y4yS* mutant strain shows higher competitiveness for nodulation on *Lotus tenuis* cv. Esmeralda than the wild type strain, as it was previously observed for a mutant affected in the T3SS functionality. The product encoded by *y4yS* is a membrane protein. Its absence affects secretion through T3SS. The inability of the y4yS mutant to secrete NopA and NopX proteins may be due to a role contributing to the structure or secretion regulation of T3SS. Secretion analyses alone could not determine if Y4yS is a pili chaperone, a secretion regulator or a protein involved in T3SS structure assembly.

A number of observations led us to examine the effect of the *y4yS* mutation on RhcC2 levels in the cell: (1) Y4yS shared characteristics with membrane proteins involved in secretin complex formation such as the lipobox sequence and the TPR domain, (2) Y4yS shared certain homology with the Tad docking protein, (3) Y4yS also showed closer evolutionary relationship with TadD than with class V chaperones and other TPR proteins, (4) the fact that *Mesorhizobium loti* secretin RhcC2 originated from the Tad locus, and (5) our discovery of a coevolutionary relationship between the TPR secretin/pilotin or docking protein and RhcC2/Y4yS proteins. We found that the absence of Y4yS negatively affects RhcC2 levels in the cell. Future analyses will determine if this results from an effect on production or stability of RhcC2. Y4yS was localized in OM (in addition to its inner membrane localization) and *y4yS* mutation affects RhcC2 levels in membranes. Since some secretin proteins require an OM lipoprotein (pilotin or docking protein) for stabilization or membrane insertion, we here propose that Y4yS may have this role for the RhcC2 secretin of *M. loti* and be a membrane protein relevant for the structure assembly of *M. loti* MAFF303099 T3SS. For the Tad system in Aggregatibacter, where secretin is not observed in a TadD mutant strain, the loss of stabilizing physical interactions between these two transport system components may account for the abundance defect observed.

Since T3SS pilotins have not been shown to harbor TPR domains, our results could represent the first report of a pilotin-like protein with TPR domains in T3SS complexes. *M. loti* secretin RhcC2 and Y4yS have homologs in Rhizobium sp. NGR234, *B. japonicum* USDA 110, and *S. fredii*. Thus, the present results may be extensive to these three strains of rhizobia.

### Conflict of interest statement

The authors declare that the research was conducted in the absence of any commercial or financial relationships that could be construed as a potential conflict of interest.

## Supplementary material

Supplementary Figure 1 **Intracellular (pellet) proteins were isolated from the *y4yS* mutant containing plasmid pBBR1MCS-4 with the 3xFLAG fused Y4yS protein expressed under the *lac* promoter (constitutive in rhizobia), Proteins were separated by 10% SDS-PAGE and then immuno-blotted and probed with an anti-FLAG antibody**. Positions of size markers loaded onto the gels are labeled (in kDa). ± N indicate bacterial culture in the presence or absence of naringenin. Bacteria contain plasmid pMP2112.

Supplementary Figure 2 **Alignment between the aminoacid sequences of TPR proteins using MUSCLE v(3.8.31) (Edgar, 2004)**.
