# Supplementary figures and images for: The absence of protein Y4yS affects negatively the abundance of T3SS Mesorhizobium loti secretin, RhcC2, in bacterial membranes
Source: Front Plant Sci. 2015 Jan 30;6:12. doi: 10.3389/fpls.2015.00012 (PMC4311626; doi:10.3389/fpls.2015.00012)

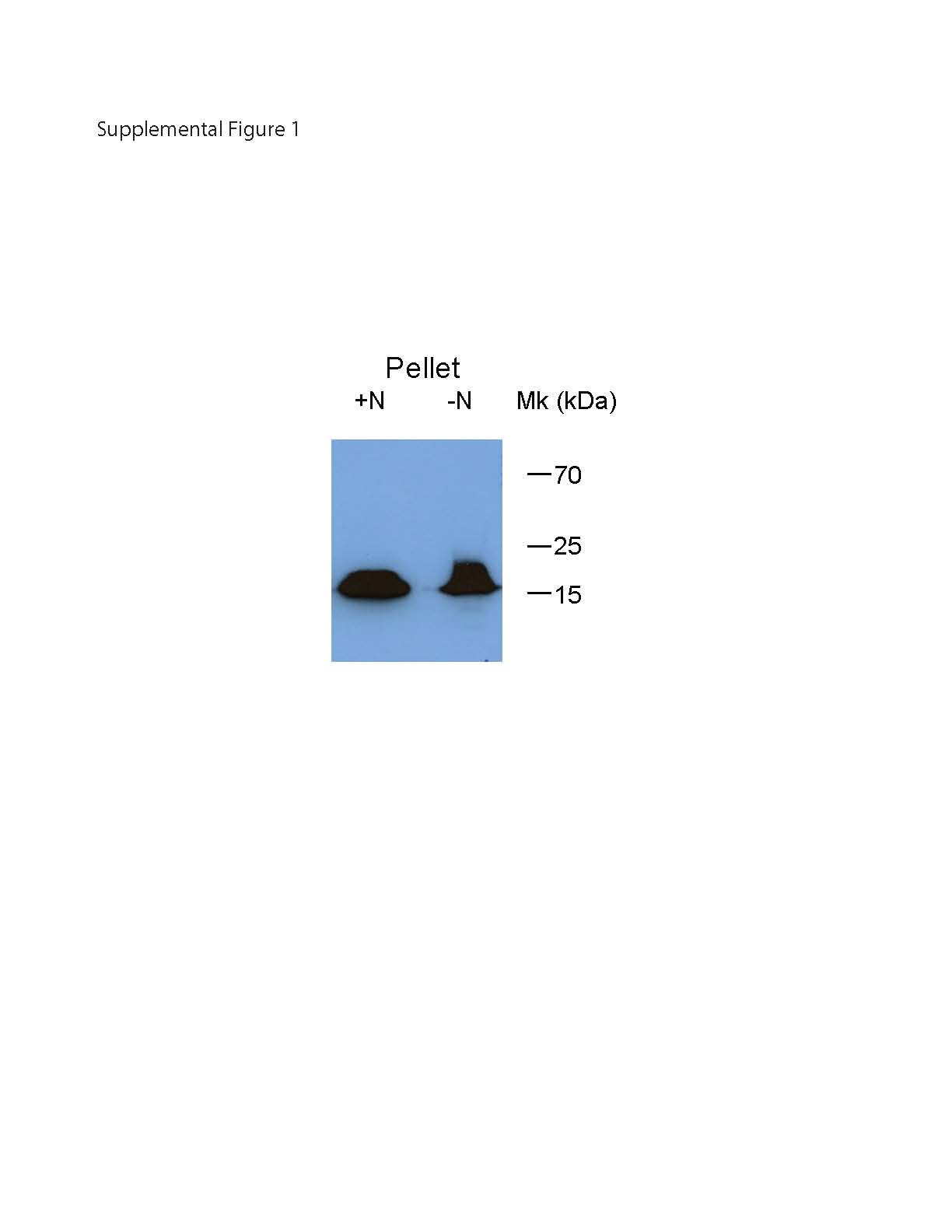

Supplement: Supplementary Figure 1 — Intracellular (pellet) proteins were isolated from the y4yS mutant containing plasmid pBBR1MCS-4 with the 3xFLAG fused Y4yS protein expressed under the lac promoter (constitutive in rhizobia), Proteins were separated by 10% SDS-PAGE and then immuno-blotted and probed with an anti-FLAG antibody. Positions of size markers loaded onto the gels are labeled (in kDa). ± N indicate bacterial culture in the presence or absence of naringenin. Bacteria contain plasmid pMP2112. [file Image1.JPEG]
