# Supplementary material for: The absence of protein Y4yS affects negatively the abundance of T3SS Mesorhizobium loti secretin, RhcC2, in bacterial membranes
Source: Front Plant Sci. 2015 Jan 30;6:12. doi: 10.3389/fpls.2015.00012 (PMC4311626; doi:10.3389/fpls.2015.00012)
Supplement: Supplementary file 3 [file Table1.PDF]

Supplemental Table 1. Bacterial strains, plasmids, and primers used in this study

	**Genotype or characteristics**	**Reference or source**
**Strains**		
***E.coli* K-12**		
DH5α-F'IQ	F´ Φ80d *lacZ*ΔM15 Δ(*lacZYA-argF*) U169 *deoR recA1 endA1 hsdR17* (r_K^- m_K+) *phoA supE44* λ^- *thi-1 gyrA96 relA1*/F´*proAB*$^+$ *lacI*^{q}*Z* ΔM15 *zzf*::Tn5[Km^r]	Woodcock et al., 1989
S17-1 λpir	λ lysogenic S17-1 derivative producing π protein for replication of plasmids carrying *oriR6K*	de Lorenzo V. & Timmis K. N., 1994
M. loti		
MAFF303099	Wild type, Nod$^+$	Kaneko et al., 2000a,b
MAFF303099 pMP2112	Wild type, Nod$^+$ and pMP2112, Sp^r	Sánchez et al., 2009
rhcN pMP2112	*rhcN*::Gm and plasmid pMP2112, Gm^r Sp^r	Sánchez et al., 2009
y4yS	*y4yS*:: Gm , Gm^r	This study
y4yS pMP2112	*y4yS*:: Gm and plasmid pMP2112, Gm^r Sp^r	This study
MAFF*y4yS* SR pMP2112	*M. loti* MAFF303099 with a chromosomal integrated copy of *mlr8765* translationally fused to 3xFLAG and plasmid pMP2112, Tc^r Sp^r	This study
y4yS Y4yS pMP2112	*y4yS* strain with *y4yS* coding sequence cloned into pBBR1MCS-4 under *lac* promoter activity and pMP2112	This study
rhcN Y4yS pMP2112	*rhcN* strain with *y4yS* coding sequence cloned into pBBR1MCS-4 under *lac* promoter activity and pMP2112	This study
MAFF*6335* SR pMP2112	*M. loti* MAFF303099 with a chromosomal integrated copy of *mlr6335* translationally fused to 3xFLAG and plasmid pMP2112, Tc^r Sp^r	This study
y4yS 6335 SR pMP2112	*y4yS* strain with a chromosomal integrated copy of *mlr6335* translationally fused to 3xFLAG and plasmid pMP2112, Tc^r Sp^r Gm^r	This study
**Plasmids**		
pGEMTEasy	pGEM®-5Zf(+) derivative, F1 replication origin. Amp^r	Promega
pGEMUp*8765*	444 bp PCR fragment corresponding to upstream *mlr8765* flanking gene cloned into pGEMT-Easy, Amp^r	This study
pGEMDw*8765*	639 bp PCR fragment corresponding to downstream *mlr8765* flanking gene cloned into pGEMT-Easy, Amp^r	This study
pGEMUpDw*8765*	PCR fragments corresponding to upstream and downstream *mlr8765* flanking genes cloned into pGEMT-Easy, Amp^r	This study
pGEMUpDw*8765*::Gm	pGEMUpDw*8765* containing a Gm resistance cassette without transcriptional terminator, Amp^r Gm^r	This study
pBBR1MCS-4	Broad-host-range cloning vector. Amp^r	Kovach M., 1995
pBBR*y4yS*	Entire *mlr8765* gene cloned into pBBR1MCS-4 plasmid in the lac promoter orientation, Amp^r	This study
pBBR*y4yS*-FLAG	*mlr8765* coding sequence translationally fused to 3xFLAG into pBBR1MCS-4, Amp^r	This study
pK18*mob*	Mobilizable derivative of pK18, Km^r	Schafer et al., 1994
pK18*mob*Tc	pK18*mob* vector containing a Tetracycline cassette, Km^r Tc^r	Sánchez et al., 2009
pK18*mob*Tc-UpDw*y4yS*::Gm	pK18*mob*Tc derivative, *mlr8765*::Gm, Km^r Tc^r Gm^r	This study
pK18-*y4yS-2*	436 bp corresponding to the 3´end of the *y4yS* coding sequence translationally fused to 3xFLAG into the pK18*mob*Tc vector, Km^r Tc^r	This study
pK18*mobmlr6335*	783 bp corresponding to the 3´end of the *mlr6335* coding sequence translationally fused to 3xFLAG into the pK18*mob* vector, Km^r	This study
pK18*mobmlr6335*::Tc	783 bp corresponding to the 3´end of the *mlr6335* coding sequence translationally fused to 3xFLAG into the pK18*mob* vector with Tc cassette cloned, Km^r Tc^r	This study

pMP2112	IncW, contains nodD from *R. leguminosarum* bv. *trifolii* Sp^r	López-Lara et al., 1995
pBAD24 3xFLAG	Region encoding 3x FLAG epitope in pBAD24, Amp^r	Spano et al., 2008
pBAD-*y4yS*-1	*mlr8765* coding sequence translationally fused to 3xFLAG into the pBAD3xFLAG vector, Amp^r	This study
pBAD-*y4yS*-2	436 bp corresponding to the 3' end of the *mlr8765* coding sequence translationally fused to 3xFLAG into the pBAD3xFLAG vector, Amp^r	This study
**Primers**		
**Primers for mutant construction**		
*mlr8765*UpF	5' -aagcttccgctatgccaacacaga- 3'	This study
*mlr8765*UpR	5' -ggatccccgtctgtattctggtgt- 3'	This study
*mlr8765*DwF	5' -ggatcccttacgaatgctttggtc- 3'	This study
*mlr8765*DwR	5' -tctagaccgcttcttgcctttgat- 3'	This study
**Primers for complementation**		
mlr8765UpComp	5' -aagcttaattggaaaccaagca- 3'	This study
*mlr8765*DwComp	5' -ggatccaggtccggacttccttta- 3'	This study
**Primers for amplification of regions used in translational fusion to 3x FLAG analysis**		
mlr8765-FlagUp	5' -acggatccacgtgatagcactctatg- 3'	This study
mlr8765-FlagDw	5' -ttccatggaaaacgcaagcgacag- 3'	This study
mlr8765Up	5´-acggatccgccatgctgcctctcg-3´	This study
mlr6335-FlagUp	5´-acggatccgcgatgtccggtgaaact-3´	This study
mlr6335-FlagDw	5´-ttccatggattgaattatgaagccgc-3´	This study
