# Supplementary material for: The absence of protein Y4yS affects negatively the abundance of T3SS Mesorhizobium loti secretin, RhcC2, in bacterial membranes
Source: Front Plant Sci. 2015 Jan 30;6:12. doi: 10.3389/fpls.2015.00012 (PMC4311626; doi:10.3389/fpls.2015.00012)
Supplement: Supplementary file 4 [file DataSheet1.ZIP › 104728_Lepek_Table_3.PDF]

Supplementary text 1

Close blast hits to mlr6335 gene (rhcC2 gene)

>499216423|Mesorhizobium.loti_Mesorhizobium.loti
MQDQGAPRAASNSIDGTLNLSSSLGKTVHLPAPATTIFVADPTIADYQAASNTTIFVFGKKSGRTSLFALDDKGEALAAL
RIVVTQPIEELRAMLMDQVGDSSIQVSYTPRGAILSGTAPNAEVADTAKRVTEQYLGDGAQVVNNIKVAGSLQVNLSVRV
AEVSRSAMKALGVNLSAFGQIDNFRVGLLSGGGTGSGAAQGGGTAGIGFNNGAVNIGAVLDALAKEHIASVLAEPNLTAM
SGETASFLAGGEFPIPVLQENKQVSVEFRHFGVSLEFVPTVLNNNRINIHVKPEVSELSSQGAVQINGISVPAVSTRRAD
TVVELASGQSFAIGGLIRRNVNNNVSAFPWLGEMPILGALFRSSSFQKEESELIILVTPYIVKPGSSPNQMSAPTDRMAP
ALDDPPADPPRGRAAARTGAPGAKRGRGFIIQ
>496150264|Mesorhizobium.metallidurans_Mesorhizobium.metallidurans
MSRLLYAVILSYPVSAPAQNIEDKGTPPAASNSLDGTLNLSSSLGKTVHLPAPATTIFVADPTIADYQAASNTTIFVFGK
KSGRTSLFALDEKGEALVALRIVVTQPIEELRAMLSDQVGDSSIHVSYTPRGAILSGTAPNAEVADTAKRVTEQYLGDGA
QVVNNIKVAGSLQVNLSVRVAEVSRSAMKALCVNLSAFGQIDNFRVGVLSGSGAGSGAALGGGTAGIGFDNGVVNIGAVL
DALAKEHIASVLAEPNLTAMSGETASFLAGGEFPIPVLQENKQVSVEFRHFGVSLEFVPTVLNNNRINIHVKPEVSELSS
QGAVQINGISVPAVSTRRADTVVELASGQSFAIGGLIRRNVNNNVSAFPWLGEMPILGALFRSSSFQKEESELIILVTPY
IVKPGSSPNQMSAPTDRMAPALDDSPANPPRGRAAARTGAPGAKRGRGFIIE
>652900349|Mesorhizobium.WSM2561_Mesorhizobium.WSM2561
MKIVPTRHSSANRQPILIGGPRPTVSPYLGHLLCALILLSPLSVAAQDKQDERAPRAASNSIKDTLNLSSSLGKTVHLPA
PAATIFVADPTIADFQAPSNQTIFVFGKNSGQTSLFALDDNGEALAELRIVVTQPIEELRAMLKDQVGDYSIEVSYTPRG
AILSGTAPDAEVADTAKRVTEQYLGDGAQVVNNIKVAGSLQVNLSVRVAEVSRSAMKALGVNLSAFGQIDNFRVGLLSGA
GAGSGAAQGGGTAEIGFNNGAVKIGAVLDALAKEHIASVLAEPNLTAMSGETASFLAGGEFPIPVLQENGQVSVEFRHFG
VSLEFVPTVLSNNQINIRVKPEVSELTSQGAVQVNGISVPAVSTRRADTVVELASGQSFAIGGLIRRNVNNNVTAFPWLG
EMPILGALFRSSSFQKEESELIILVTPYIVRPGSSPNEMSAPTERMAPTLDGGGTPTNSPASPPRGRAAARTGSPGAKAG
LGFIIE
>657243377|M.ciceri_M.ciceri
MLILQHPCPALNWRAIAIKVPRPAVPRYLGHLLCALIVTFPLGVAAQNKQDKGAPRAASNSINATLTLSSSLGETVHLPA
PATTIFVADPTIADFQAPSSKTIFVFGKKSGRTSLFALDGNGEPLAELRIVVTQPIGDLRAMLREQVGDYPIRVNYTPRG
AILSGTAPDAEVADTAKRVTEQYLGDGAQVVNNIKVAGSLQVNLSVRVAEVSRSAMKSLGVNLSAFGQIGNFKVGVLSGS
GASAGSGSTQGGGTAEIGFDNGVVNVSAVLDALAKEHIASVLAEPNLTAMSGEKASFLAGGEFPIPVLQENRQVSVEFRH
FGVSLEFVPTVLSNNQINIHVTPEVSELSTQGAVQINGISVPAVSTRRADTVVELASGQSFAIGGLIRRNVNNDVRAFPW
LGEMPILGPLFRSSSFQKEESELVILVTPYIVRPGSNPNQMSAPTERAAPALNGGGAPTNSVASPPRDRAAIRAGAPSAQ
GGLGFIIE
>504670442|Sinorhizobium.fredii_Sinorhizobium.fredii
MKATPTGHASADRWPTPIVGSRPTVPYWSNLLYALVLLSPMSAAAENNGDEAVPRAAPNSINATLNLSSSLGKTVHLPAP
AATIFVADPTIADYQAPSNRTIFVFGKKFGRTSLFALDENGEALAELHVVVTQPIGDLRAMLRDQVGDYPIHVSYTPRGA
ILSGTAPNAEVVDIAKRVTEQFLGDGAPIVNNIKVAGSLQVNLSVRVAEVSRSSLKALGINLSAFGQFGNFKVGVLNRGA
GLGSATGSGGTAEIGFDNDAVSVGAVLDALAKEHIASVLAEPNLTAMSGETASFLAGGEFPIPVLQENGQTSVEFRRFGV
SLEFVPTVLDNNLINIHVKPEVSELSLQGAVQVNGIAVPAVSTRRADTVVELASGQSFVIGGLIRRNVNNDISAFPWLGR
IPILGALFRSSSFQKEESELVILVTPYIVRPGSNPNQMSAPTDRMAPALGTPPRARAAISTDAPSVKGDLGFIIE
>651622971|Ensifer.TW10_Ensifer.TW10
MKAIPTGHGSADRWPTLIAGPGPTVPYWSSLLCVLVLLSPMSAVAEYNGDKAVPRAAPNSINATLNLDSSLGKTVHLPAP
AATIFVADPTIADYQAPSNKTIFVFGKKFGRTSLFALDENGEALAELRVVVTQPIADLRAMLRDQVGDYPIHVSYTPRGA
ILSGTAPNAEVVDTAKRVTEQFLGDGAPVVNNIKVAGSLQVNLSVRVAEVSRSAMKALGINLSAVGQIGTFKVGLLNRDA
GLGSATGGGGTAEIGIDNGAVNVSAVLDVLAKEHIASVLAEPNLTAMSGETASFLAGGEFPIPVLQENGQTSVEFRRFGV
GLEFVPTVLDNNLINIHVKPEVSELSSQGAVQSNGISVPAVSTRRADTVVELASGQSFAIAGLIRRNVNNDISAFPWLGR
IPILGALFRSSSFQKEESELVILVTPYIVRPGSSPNRMSVPTDLMAPPLDRATTPPRGDATASTDSPGGTGDLGFIIE
>496113950|Mesorhizobium.alhagi_Mesorhizobium.alhagi
MKIVPTRHPGANRQPILIDGPRPTVSPYLGHLLCALILLSPHSAAAQNSGDDGATRAASNSINATLNLSSSLGKTVHLSA
PAATIFVADPTIADYQAPSNKTIFVFGKKSGQTSLFALNDNGEALAQLRIVVTQPIGDLRAMLRDQVGDYPIHVSYTPRG
AILSGTAPDAEVVDTAKRVTEQFLGDGAQVVNNVKVAGSLQVNLSVRVAEVSRSAMKELGINLSAAGQIGNFKVGLLRGG
RGAGSGAANGGGTAEVGFDDGNISVGAVLDALAKEQIASVLAEPNLTAMSGETASFLAGGEFPIPVPQENGQVSIEFRRF
GVSLEFVPTVLNNNQINIRVKPEVSELTSQGAVQINGISVPAISTRRADTVVELASGQSFAIGGLIRRNVSNNIRAFPWL
GEVPILGALFRSSSFQKEESELVILVTPYIVRPGSTPNQMSAPTDRMGQPLDGGATLTNSLASPPRDRVGAPGAKGGLGF
IIE
>652916183|Mesorhizobium.WSM3224_Mesorhizobium.WSM3224
MKIVPIRHAELNRRSNLVDGSRATGGPYLGPLLCALMLLSPLSAAAQGNGGEATPRAASNSINATLSLSSSLGKTVSLPA
PAATIFVADPTIADYQAPSNKTIFVFGKKSGRTTLFALNDKGEALASLQIVVTQPIGDLRAMLRDQVGDYPIRVKYTPRG
AILSGTAPDAETVDAAKTVTEQFLGEGAQVVNRIKVAGSLQVNLSVRVAEVSRGAMKELGINLSALGQIGNFKVGLLSGG
GRGAGSGAANGGGTAEIGYDDGNVSIGAVLDALAKEHIASILAEPNLTAMSGETASFLAGGEFPIPVPQENGQVSIEFRH

FGVSLEFLPTVLNNNQINIRVKPEVSELTSQGAVQINGISVPAISTRRADTVVELASGQSFAIGGLIRQTVSNNISAFPG
LGQVPILGALFRSSSFQKEESELVILVTPYIVRPGSSPDQMSAPTDRMASPSDGGKPRSNVRRPPGGRAATHAGAPSAKS
SVGFVIE
>493227142|Mesorhizobium.amorphae_Mesorhizobium.amorphae
MTALNIVRKNHSGGDRRSIVINTLKSGVFPCLRHFLCAVILLSPLSAVAQIKQDDSGETSHASPNSVSGTLNLSSSLGKT
VHLSAPAASIFVADPTIADYQAPSNKTIFVFGKKSGRTTLFALDENGEALAQLQIVVAQPIEDLRAMLRAQTGDYPIHVS
YTPRGAVLSGTAPNAEVVDTAMKITEQFLGAGAQIVNKIQVTGSLQVNLSVRVAEVSRSAMKKLGVNLSAFGQIGNFKVG
LLSSGNEAGFDSGNKAGIGFSDGNINVSAVLDALATEHLASVLAEPNLTAMSGESASFLAGGEFPIPVVQNNGQASVEFR
HFGVSLEFVPTVLSNNQINIRVKPEASELSSQGAVQMNGISVPAISTRRADTVVELASGQSFAIGGLIRRSVNTDISAFP
WLGDVPVLGALFRSSSFQKEETELVIIVTPYIVRPGSSPSQMSAPTDRIGSPLGVGGTPTNSLATPPPSHGAPRPSVSSR
AGGAGFIIE
>685098467|Mesorhizobium.plurifarium_Mesorhizobium.plurifarium
MVSPCPAYLLCALILFFPFCATAQNKRYEGAPRATANSINGTLNLSSSLGKTIHLSAPAASIFIADPTIADFQAPSNKTV
FVFGKKSGRTSLFALDANGEALAQLHIVVTEPIEDLRAMLRTRVGDYPIHVSYTPRGAILSGTAPNAQVVDTATKVTEQF
LGDGAQVVNEIQVAGSLQVNLSVRVAEVSRSAMKELGINLSAFGKIGNLTVGLASGQGAGSGAASGGDMAGVAYNDGNVN
LGAVLDALAKEHVASVLAEPNLTAMSGESANFLAGGEFPIPVVQGNGQVSVEFRHFGVGLEFVPTVLNNEQINIRVKSEA
SELSTQGAVQINGISVPAVSTRRADTAVELGSGQSFAIGGLIRRNVNADIRTFPWLGDVPILGALFRSSSFQKEETELVI
IVTPYIVRPGSNPKQMSAPTDRLGAASDVEATPMNSSENPPQDHATGGSGALDGFIIE
>685083534|Mesorhizobium.SOD10_Mesorhizobium.SOD10
MVSPCPAYLLCALILFFPFCATAQNKRYEGAPRATANSINGTLNLSSSLGKTIHLSAPAASIFIADPTIADFQAPSNKTV
FVFGKKSGRTSLFALDANGEALAQLHIVVTEPIEDLRAMLRTRVGDYPIHVSYTPRGAILSGTAPNAQVVDTATKVTEQF
LGDGAQVVNEIQVAGSLQVNLSVRVAEVSRSAMKELGINLSAFGKIGNLTVGLASGQGAGSGAASGGDMAGVAYNDGNVN
LGAVLDALAKEHVASVLAEPNLTAMSGESANFLAGGEFPIPVVQGNGQVSVEFRHFGVGLEFVPTVLNNEQINIRVKSEA
SELSTQGAVQINGISVPAVSTRRADTAVELGSGQSFAIGGLIRRNVNADIRTFPWLGDVPILGALFRSSSFQKEETELVI
IVTPYIVRPGSNPKQMSAPTDRLGAASDVEATPMNSSENPPQDHAPGGSGALDGFIIE
>654899165|Bradyrhizobium.elkanii_Bradyrhizobium.elkanii
MKIALDNEGGSDRRSIVASALWSAVCPSLSHLLCAVALLSPLAAAAQTTRDARGGPPRAALSSINGTLDLSSSLGKTIHL
PGPAASIFVADPTIADYQAPSNTTIFVFGKKSGRTSLFALNDNGEALAELRVVVTQPIEDLRAMLKAQVGDYPIQVSYTP
RGAILNGTAPNADIVATAVKVTEQFLGPGALVVNKIQVAGSLQVNLSVRVAEVSRSAMKELGINLSAMGQNGTFFFSSGK
GAGSGTASGGGKAGIGFSAGSINIGAVLDALASEHLASVLAEPNLTAMSGESASFLAGGEFPIPVMQDNRQVSVQFRHFG
VSLDFVPTVLNNNQINVRVKPEVSEISKEGEVKVNGMAVPALSTRRAETVIELGSGQSFAIGGLIRRNFSTDISTFPWLG
DLPILGALFRSSSFQKQETELVIIVTPYIVRPASNPNRMSAPADRIGPPSDLGRTLTNTLASPPRGRDAPRTSVPGATGG
AAFIIE
>685092163|Mesorhizobium.ORS3324_Mesorhizobium.ORS3324
MVSPCPAYLLCALILFSPFSATAQDKRYEGAPRATANSINGTLNLSSSLGKTIHLSAPAASVFIADPTIADFQAPSNKTV
FVFGKKSGRTSLFALDANGEALAQLNIVVTEPIEDLRAMLRTRVGDYPIHVSYTPRGAILSGTAPNAQVVDTATKVTEQF
LGDGAQVDNEIQVAGSLQVNLSVRVAEVSRSAMKELGINLSAFGKIGNLTVGLASGKGGGSGAASGGGTAGVAYNDGNVS
LGAVLDALAKEHVASVLAEPNLTAMSGESANFLAGGEFPIPVVQGSGQVSVEFRHFGIGLEFVPTVLNNEQINIRVKSEV
SELSTQGAVQINGISVPGVSTRRADTAVELGSGQSFAIGGLIRRNVNTDIRAFPWLGDLPILGALFRSSSFQKEETELVI
IVTPYIVRPGSNPKQMSTPTDRLGSASDVGPTPMNSSENPPQDHATGGSGVLDGFIIE
>685103604|Mesorhizobium.ORS3359_Mesorhizobium.ORS3359
MLKAKIALKVVPNGHSPANRRSTVIDGLRTMVSPCPAYLLCALILFSPFSATAQDKLYEGAPRATANSINGTLNLSSSLG
KTIHLSAPAASVFIADPTIADFQAPSNKTVFVFGKKSGRTSLFALDANGEALAQLNIVVTEPIEDLRAMLRTRVGDYPIH
VSYTPRGAILSGTAPNAQVVDTATKVTEQFLGDGAQVDNEIQVAGSLQVNLSVRVAEVSRSAMKELGINLSAFGKIGNLT
VGLASGKGGGSGAASGGGTAGVAYNDGNASLGAVLDALAKEHVASVLAEPNLTAMSGESANFLAGGEFPIPVVQGSGQVS
VEFRHFGIGLEFVPTVLNNEQINIRVKSEVSELSTQGAVQINGISVPGVSTRRADTAVELGSGQSFAIGGLIRRNVNTDI
RAFPWLGDLPILGALFRSSSFQKEETELVIIVTPYIVRPGSNPKQMSTPTDRLGSASDVEPTPMNSSENPPQDHATGGSG
VLDGFIIE
>528843909|Rhizobium.etli_Rhizobium.etli
MKLVSDLYATTHQRSSSGGGLGASVLSCCAYFLCALILFFPFSATAQNDQNKEASHATHNTINGTLDLSSSLGKTIHLPA
PAASIFIADPTIADFQAPSNKTIFVFGKKSGRTSLFALDDNGEVLAELRVVVTESIEDLRAMLRARVGDYPIHVRYTPRG
AILSGTAPNAQVVETATKVTEQFLGDGAQVVNEIEVSGSLQVNLSVRVAEVSRSAMKELGINLTAVGTIGNLTVGFSSGK
DSGAASGGGTAGVAYNDWNTNLGAVLDALAREHVASVLAEPNLTAMSGESANFLAGGEFPIPIARDNGQVSIEFRHFGVG
LEFLPIVLNNDQINIRVKSEASELSKQGAVQISGISVPAISTRRAETVVELGSGQSFAIGGLIRRNVSADIRAFPWLGEV
PILGALFRSSSFQKEETELVIIVTPYIVRPGTNSKQMSAPTDRLGPPLGPRATPTNSPQDATRRDGGSGALGGFIIE
>608610147|Bradyrhizobium.DOA9_Bradyrhizobium.DOA9
MAASSSHSAAFPRLCYALCAVALLFPLAAAAQVKRDGKGEAPRAAPGSTTGTLNLTSSQGKTVHLTGPAASVFIADPTIA
DYQAPSNTTIFVFGKKSGRTSLFALNDNGEALAELRVVVTQPIEDLRATLKAEVGDYPIQVSYTPRGAILSGTAPNAEVV
ETARKVTEHFLGAGALVANKIQVAGSLQVNLSVRVAEVSRTAVKDLNINFTASSPNGAFLVTGKGGGSGAAGGGGTIGIG
FSAGHTNLSAVLDALASEHLASILAEPNLTAMSGEAASFLAGGEFPIPVMQDNRQVSVQFRQFGVSLEFVPTVLNNNQIN
VRVKPEVSELSSEGEVKINGIAVPALSTRRASTVVELASGQSFAIGGLIRRNFNTDIGEFPWLGDVPILGALFRSSSFQK
RETELVIIVTPYIVRPGPNPNRMSAPSDRISPPSDAGRILTNTVARPPRERDAPRASAPGLTGSSGFIIE
>653487231|Bradyrhizobium.Cp5.3_Bradyrhizobium.Cp5.3

Supplementary text 1

MKFTRSRSIDRRSGAVTSDILLRILYALCTVALVFPLTAAAQIKRDASQGAPRATPGSLNGTLNLTSSQGKTVHLPAAAA
SIFVADPTIADYQAPSNTTIFVFGKKSGRTSLFALNDNGEALAELRVVVTQPVEDLRAALKAEIGDYPIQVTYTPRGAIL
SGTAPNAEVVDHAKTITEQFLGAGALVVNKIQVAGSLQVNLSVRVAEVSRTAMKELGISLSASGQNGAVVFGFNSGKAGG
SGASGGGGTASIGFGVNAANVSAVLDALANEHLASVLAEPNLTAMSGESASFLAGGEFPIPVMQDNRQVSVQFRQFGISL
EFVPTVLSNNQINIRVKPEVSELSKEGQVNVNGMSLPGLSTRRASTVVELASGQSFAIGGLIGRNFNTDISTFPGLADVP
ILGALFRSSSFQKQETELVIVVTPYIVRPASTAGKMSAPTDRIRPPSDAGRTLTNTLASSPQSRSSPRKPDPGGTEGTGF
IIE
