# Supplementary material for: The absence of protein Y4yS affects negatively the abundance of T3SS Mesorhizobium loti secretin, RhcC2, in bacterial membranes
Source: Front Plant Sci. 2015 Jan 30;6:12. doi: 10.3389/fpls.2015.00012 (PMC4311626; doi:10.3389/fpls.2015.00012)
Supplement: Supplementary file 4 [file DataSheet1.ZIP › 104728_Lepek_Table_6.PDF]

Supplementary text 4

Set2 for mlr8765:
Close blast hits to mlr8765 gene plus 7 pilotins corresponding to TPR secretins of supplementary text 3.

```
>499216440|Mesorhizobium.loti_Mesorhizobium.loti
MNSHQNTDGRAFRNSLFFSVLAMLPLGGCASLDKPALSVRETSSPELLNANQIDPAMRERILSAVGQDAQERALRDELTQ
HPDNVDAAIRLTNALVAQKRPHEALQVLDRLLVAAPGNLRALNAKGVILDLEGRHDAAQVLYRQALETEPGNQMVQHNLD
LSLAF
>496150295|Mesorhizobium.metallidurans_Mesorhizobium.metallidurans
MLPLGGCASLDRPALSVKETSSPELLNAKQIDPAMRERILRAVGQDAQERALRDELKQHPDNVDAAIRLTNALVAQKRAH
EALHVVDTVLVAAPGNLRALNAKGVILDLDGRHDAAQVLYRQALETEPGNEMVQHNLNLSLTL
>657243333|M.ciceri_M.ciceri
MKFVRNTVSPSPMFLRRSSLLFAVFAILPLGGCASWQKPALSVQETSSPELLSADQIDPAMRERILREVGQDVQERALRD
EVKQHPDNVDAAIRLTKALVAQKRPHEAVEVLDSVLVAAPANVRALNAKGVILDIEGRHDAAQALYRRALETEPGNQMVQ
HNFNLSLALTASPNSQG
>652900438|Mesorhizobium.WSM2561_Mesorhizobium.WSM2561
MKPNENIVPTLMPFRRYSLLYAVLAILLLGGCATDKSALSVKETSSPELLNANQIDPAMRERILRAVGQDAEERALRDEL
KQHPDNIDAAIRLTNALVAQNRAHEALQVVDSVLVAAPGNLRALNAKGVILDIEGRHNAAQALYRQALETEPGNEMVRHN
FNLSRAIAGKSDRGR
>651622891|Ensifer.TW10_Ensifer.TW10
MNSHENTVAAPLLSFRLTLVLFAVFAILPLGGCASWDKPVLSVKETSTPQLLGANQIDAAMRQRILRAVGQDAEERASRD
DLKRHPRNVDAAIRLTKALVAQKRPHEALPALDNVLVAAPENLRALNAKAVILDIEGRHDAAQELYRKALETAPENEMLH
HNLNLSLAYAGKSEQSSLPQSR
>505446299|Sinorhizobium.fredii_Sinorhizobium.fredii
MNSHENRVAAPLLSFRLNLVLFAVLSVLPLGGCARWDNPVLSVKETSAPQLLGANQINAATRQRILRAVGEDAQERALRD
DLKQHPGNVDAAIRLTKALVAQKRPHEALQVLDNVLVVTPDNLRALNAKAVILDIEGRHDAAQELYRQALETNPENQMLH
HNLNLSLAFEGKSEQSTLPQSR
>528843840|Rhizobium.etli_Rhizobium.etli
MGIAVALLWRSSRLASVLFAILAIVPLTGCASWGKQALSVKETPPPELLGAKDIDPLMRKRILSAVDEDVRERALRDELE
RQPDNVDAAIRLAKALLAQKRPKEALEVLDRVLLTAPGNLRALNAKGVVLDIEARHDAAQALYRQALENEPGNQMLLNNL
NRSLALDGKSGPNAPAGSQ
>493217423|Mesorhizobium.amorphae_Mesorhizobium.amorphae
MKSYQNLHAIRPRSFRLASIVFAILTIVPLSACSSWNKSGLSVKEAAPPDLLGAKDIDPAMRERIARAVGPDTDERALQD
ELKQQPGNVDAAIRLTKALVAQKRESEALQILDNVLLAAPNNLRALNAKGVVFDLEGRHDAAQALYRQALKTEPGNQMLR
NNLNLSLALDGTVEPSASARTL
>496113926|Mesorhizobium.alhagi_Mesorhizobium.alhagi
MKETSPPELLGANHIDPAMRERILRAVGQDSQERALRDELKQHPDNVDAAIHLAKALLAQERPHEALQVLDNVLVAVPGN
LRALNAKGVVLDVEGRHDAAQALYRQALESEPGNQMLHHNLNLSLAFDRKPERNALAQSR
>652914351|Mesorhizobium.WSM3224_Mesorhizobium.WSM3224
MNLRENTIAAPLLSFRLNPVLFAALAILPLGGCASWNKPDLSVKETAPPALLGADHIDPAMRERILRAVGQDSQERALRD
LKQDPANVDAAISVTKLLLAQKRQQEALQVLDRVLVADPDNLRALNAKGAVLDSQGQHEAAQALYLKALKAEPGNQMLQH
NLDLSLASGGKSEPSAL
>685083508|Mesorhizobium.SOD10_Mesorhizobium.SOD10
MKETPSPELLGAKDINPAMRERILTSVGEDADERALRDQLQQEPGNVDAAIHLTKALVAKKRPKEALQVLDGVLIAAPGN
LRTLNAKAVVLDICGRHGAAQALYRQALRKEPGNQMLVNNLNRSLALDEKSGRSAPARSG
>685112787|Mesorhizobium.plurifarium_Mesorhizobium.plurifarium
MKETPSPELLGAKDINPAMRERVLSSVGQDAHERALRDQLQQQPGNVDAAIELTKALLARKLPKEALQVLDGALIAAPGN
LRTLNAKAVVLDICGRHGAAQALYRQALRKEPGNQMLVNNLNRSLALDEKSGRSAPARSR
>654899141|Bradyrhizobium.elkanii_Bradyrhizobium.elkanii
MNSLTTTFAARLYAFHPPSGLTLLALFATLALGGCASLDHQAISVQETPRPELLGAKDIDPAMRERIAHALLRVSGEESL
REALKQKPDNVDAAISLTQALLAQRRAGEALEVADKILLTVPGDLRAMNAKGVVLDAEGRHDEAQALYREALAAAPGNQM
LRNNLGLSLALARNANTGHASLQPLSHEPHALAGSP
>685103569|Mesorhizobium.ORS3359_Mesorhizobium.ORS3359
MLGAKDINPGMRERILSSVGQDAHERALRDQLQQQPGNVDAAIELTKALLARKLPNEALQVLDGVLIAAPGNLRTLNAKG
VVLDICGRHGAAQALYRQALRKEPGNQMLVNNLNRSLALDEKSGRSAPARSR
>685092192|Mesorhizobium.ORS3324_Mesorhizobium.ORS3324
MLGAKDINPGMRERILSSVGQDAHERALRDQLQHQPGNVDAAIELTKALLARKLPNEALQVLDGVLIAAPGNLRTLNAKG
```

```
VVLDICGRHGAAQALYRQALRKEPGNQMLVNNLNRSLALDEKSGRSAPARSR
>653487193|Bradyrhizobium.Cp5.3_Bradyrhizobium.Cp5.3
MKPYQMNACRRLCSRSPHLCLAVLAILLLGGCTSRDKPALSAQQITPPELLNTKEIDAATRERFAYALRGDVDEEALPDA
LNKQPDNVNAAIPLARALLARKCPDRALEVLDNVLLAAPSDLRILNAKGVVLDHEGRHHEAQALYRQALAMAPGNPMLTN
NLKLSLALDEKDKAGSASLQPLSDSPNESVQ
>640610459|Bradyrhizobium.DOA9_Bradyrhizobium.DOA9
MNLCKINFLRRWPNFRRHSCSAILPMLATVLLAGCVNSHKSGFSQQPTSAAELVGAKEVDPAMRERIALALGRDADERAL
RDALKQRPDDVDAAIPLARALLERKCPNDALEVLDGILLAAPGDLRALNAKAVVLDHEGRHREAQELYRQALAAEPANPM
LRNNFKLSLALEGKTETGGANPAPQADGPHFAALSRTSPCGSGSEW
>H7C7V6|BRADU_BRADU
MKSNDTSFRRYLCSASPRLRPALLAMLTVVLLAGCATADKHGLSPQQRSASDLADSEEID
PAARERISHALGRDADEGALRDALKQQPNNIDAAIPLARALLARNCPNDALEVLDGVLLA
TPGDLRALNAKAVVLDHEGRHQEAQELYRQALAAEPANPMLRNNFGLSLALQGKMDAGDA
SAAPQAGSRKALARSR
>Q7X0L5|AGGAC_AGGAC
MYSKSLKNVLLCSMILSVTACSTVLNKRPLSADKITAKETLYQSTNNNDALVAMYRSVLK
DKEDPITRYKLSEIYYKKGDSNSSLLYLKPLLTNGGQLMEKAKILQARNLNQLKRYQEAL
EVENSLLVSSPKNGEVYNLRGVTYALMGNPNKANEDINKAREYFLNDAVAVNNMAMLSII
NGDYRNAVSLLLPQYLNGVREQRLVHNLVFALVKNNEIDYAKDIIVKENINTSPDDLVNA
LKKTDRMSSNITR
>Q989C4|RHILO_RHILO
MTRPMRLAAVAAACLTMALAGCQTNGAGSTDGVVRTNEPSKGDVTSFGDAFDGLKTVSDV
EYYASDQAVAEATNQFRAENYGNAGALFFKATQLAPNDGGAWMGLAASCDRIRRFDLADR
AYGKAFKLVGASAEYYNNVGYSYLLRGKLQDARTSFLKAYELAPNDPTVANNLKLLSSSV
QNIER
>Q9HXJ2|PSEAE_PSEAE
MTVRAALVFLLAVGLTGCVTSGDQNPLKTDKGRDEARDAYIQLGLGYLQRGNTEQAKVPL
RKALEIDPSSADAHAALAVVFQTEMEPKLADEEYRKALASDSRNARVLNNYGGFLYEQKR
YEEAYQRLLEASQDTLYPERSRVFENLGLVSLQMKKPAQAKEYFEKSLRLNRNQPSVALE
MADLLYKEREYVPARQYYDLFAQGGGQNARSLLLGIRLAKVFEDRDTAASYGLQLKRLYP
GSLEYQEFQAEK
>Q7CJM8|YERPE_YERPE
MKLTKLWRVCLVVSVLTGCSGTPPENTSQAVAGQTRLQLGLAYLAQGDLTAARKNLEKAV
EADPQDYRTQLGMAFYAQRIGENSAAEQRYQQAMKLAPGNGTVLNNYGAFLCSLGQYVSA
QQQFSAAALLPDYGQVADSLENAGYCFLRANQDKQARVLLSRALKYDPDKGEPLLAEAQR
HFGEGNRAQAQLLLDVYQHTLPASAESLWLQIRFAALAGRQDSVQRYGKQLARSFPQSKQ
YQHFLANEY
>C9WZ30|NEIM8_NEIM8
MPFKPSKRISLLLVLALGACSTSYRPSRAEKANQVSNIKTQLAMEYMRGQDYRQATASIE
DALKSDPKNELAWLVRAEIYQYLKVNDKAQESFRQALSIKPDSAEINNNYGWFLCGRLNR
PAESMAYFDKALADPTYPTPYIANLNKGICSAKQGQFGLAEAYLKRSLAAQPQFPPAFKE
LARTKMLAGQLGDADYYFKKYQSRVEVLQADDLLLGWKIAKALGNAQAAYEYEAQLQANF
PYSEELQTVLTGQ
>Q1D7T6|MYXXD_MYXXD
MFRLSTASCSLALLLVSSGCSHTPTEKEKRSAEIHYDLALQAQQAGELQDALRELQVSLK
NDPDYPDANNAMGILLHLAFRRPDEAVKHYTKALEVRPDFSEARTNLANVHLDQGRYDDA
IKLYELVLNDMLYPTPFIAQGNLGWAYYKKGEPDRAVESIKAAVTTNPNFCLGYKNLGLI
YDETGRTSEACRQFTHYRENCPDVAEAYMREGVCQAKLGQVDAAKAAFATCETKAKAGEQ
VLKDDCRRLLEKL
```
