# Supplementary material for: The absence of protein Y4yS affects negatively the abundance of T3SS Mesorhizobium loti secretin, RhcC2, in bacterial membranes
Source: Front Plant Sci. 2015 Jan 30;6:12. doi: 10.3389/fpls.2015.00012 (PMC4311626; doi:10.3389/fpls.2015.00012)

```
tr|TadD|Q7X0L5_AGGAC_TadD_OS_Aggregatibacter_actinomycetemcomitans_GN_tadD_PE_4_SV_1
tr|Mlr8765|Q989N3_RHILO_Mlr8765_protein_OS_Rhizobium_loti__strain_MAFF303099__GN_mlr8765_PE_4_SV_1
tr|PilF|Q9HXJ2_PSEAE_Type_4_fimbrial_biogenesis_protein_PilF_OS_Pseudomonas_aeruginosa__strain_ATCC_15692_/_PAO1_/_1C_/_PRS_101_/_LMG_12228__GN_pilF_P
tr|bll_1801|Q9ANI6_BRAJP_ID192_OS_Bradyrhizobium_japonicum_GN_id192_PE_4_SV_1
sp|y4yS|Y4YS_RHISN_Uncharacterized_protein_y4yS_OS_Rhizobium_sp.__strain_NGR234__GN_NGR_a00520_PE_4_SV_1
tr|SicA|K9NL80_9PSED_Chaperone_protein_SicA_OS_Pseudomonas_sp._UW4_GN_sicA_PE_4_SV_1
tr|PSPPH_2519|Q48IR6_PSE14_Type_III_secretion_component_putative_OS_Pseudomonas_syringae_pv._phaseolicola__strain_1448A_/_Race_6__GN_PSPPH_2519_PE_4_S
tr|PSPPH_2523|Q48IR2_PSE14_TPR_Domain_domain_protein_OS_Pseudomonas_syringae_pv._phaseolicola__strain_1448A_/_Race_6__GN_PSPPH_2523_PE_4_SV_1
tr|pilW|C9WZ30_NEIM8_Type_IV_pilus_biogenesis_lipoprotein_PilW_OS_Neisseria_meningitidis_serogroup_C__strain_8013__GN_pilW_PE_4_SV_1
tr|tgl|Q1D7T6_MYXXD_Social_gliding_motility_protein_Tgl_OS_Myxococcus_xanthus__strain_DK_1622__GN_tgl_PE_4_SV_1

                                                      .                           :                                                                      :
-----------------------MYSKSLKNVLLCSMILSVTACSTVL-NKRPLSADK---------ITAKETLYQSTNNNDALVA-MYRSVLKDKEDPITRYKLSEIYYKK-GDSNSSLLYLKPLLTNGGQLMEKAKILQARNLNQLKRYQEALEVENS 125
--------------MNSHQNTDGRAFRNSLFF-SVLAMLPLGGCASL--DKPALSVRE----------TSSPELLNANQIDPAMRERILSAVGQDAQERALRDELTQ----------------------HPDNVDAAIRLTNALVAQ-KRPHEALQVLDR 110
-----------------------MTVRAALVF---LLAVGLTGCVTSG-DQNPLKTDKGRDEARDAYIQLGLGYLQRGNTEQAKVP-LRKALEIDPSSADAHAALAVVFQTE-MEPKLADEEYRKALASDSRNARVLNNYGGFLYEQ-KRYEEAYQRLLE 130
---------------------------MADPDGLHRVAVPLAPGVSKSGDIPSISGPE-----RDLLCALSYVHLACGQSAHSLAL-LRIAAREHSNDVGLLRILAYTLIAE------------------------------------GLGEEALHVLDR 91
-----------MNSHENRVAAPLLSFRLSLVLFAVLSVLPLGGCARW--DNPVLSVKE----------TSAAQLLGANQINAATRQRILRAVGEDAQERALRDDLKQ----------------------HPGNVDAAIRLTKALVAQ-KRPHEALQVLDN 114
---------------------MSRDKQQDEQLALDVVDAVLGGAALK--DVQGISDEH-----MNGLYAFAFQFYEQGRLDDAEKF-FHFLCIYDLNNSHYWMGLAAVHQLK------------------------------------QNHQKAIDLYAI 95
---------------------------MSISQ-----------------DRECIELLH----------GMGDLYRRSGQPQRALVM-LLIAIQLAPTNSALLHSLVLAFTDS------------------------------------GDTDRAIAALDR 69
MALALKGCRRLIRLAPPPFTRALPMPPRSVPTLLLLGCLSLAACSTQ--PKSDASYER--------FMQLAADLEKRGDLTTAASF-YQRATQQPEAGVEAWRKLGAALLVS-NDTRGAERAYQRALELKADDPDALLGLGTAQLRQ-GKLERAITALTQ 147
-----------------------MPFKPSKRI-SLLLVLALGACSTSYRPSRAEKANQ----VSNIKTQLAMEYMRGQDYRQATAS-IEDALKSDPKNELAWLVRAEIYQYL-KVNDKAQESFRQALSIKPDSAEINNNYGWFLCGRLNRPAESMAYFDK 130
------------------------MFRLSTASCSLALLLVSSGCSHTPTEKEKRSAEI--------HYDLALQAQQAGELQDALRE-LQVSLKNDPDYPDANNAMGILLHLAFRRPDEAVKHYTKALEVRPDFSEARTNLANVHLDQ-GRYDDAIKLYEL 126
1.......10........20........30........40........50........60........70........80........90.......100.......110.......120.......130.......140.......150.......160
```

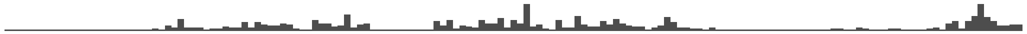

```
                  .      .    *
LL--VSSPKNGEVY-NLRGVTYALMGNPNKANEDINKAREYFLNDAVAVNNMAMLSIINGDYRNAVSLLLPQYLNGVREQRLVHNLVFALVKNNEIDYAKDIIVKENINTSPDDLVNALKKTDRMSSNITR---------- 253
LL--VAAPGNLRAL-NAKGVILDLEGRHDAAQVLYRQALETEPGNQMVQHNLDLSLAF----------------------------------------------------------------------------------- 165
ASQDTLYPERSRVF-ENLGLVSLQMKKPAQAKEYFEKSLRLNRNQPSVALEMADLLYKEREYVPARQYYDLFAQGGGQNARS-LLLGIRLAKVFEDRDTAASYGLQLKRLYPGSLEYQEFQAEK----------------- 252
LDALDTQPSSRIPMTLLRSHALRHAGRMAEAREVFQTYVSLRASTALIRKQ------------------------------------------------------------------------------------------ 142
VL--VVTPDNLRAL-NAKAVILDIEGRHDAAQELYRQALETNPENQMLHHNLHLSLAFEGKSEQRTLPQSR---------------------------------------------------------------------- 182
AF--AQGKNDYRPM-LYTGQCHLALGKVGKARLCFEYVLEQATEDDLRKQAQVYLDTLSHMEKDCSETEHE---------------------------------------------------------------------- 163
LV--EQQGESAALL-LLRSRALWKAGRKDDARQCFRRYLEARRAAQ----------------------------------------------------------------------------------------------- 112
AADALQQPQAW----NRLGIAHILSGQADAAQSAFGTSLRLAPNDLDIRCNLALAYALGDDDQKALETIRSVSQSPLAQPRHQRNQLLVMVLAGKEKDLKNMTFDDITKAERGKLIAEARRVKAIPDRAEQARELGLIDAN 284
ALADPTYPTPYIAN-LNKGICSAKQGQFGLAEAYLKRSLAAQPQFPPAFKELARTKMLAGQLGDADYYFKKYQSRVEVLQADDLLLGWKIAKALGNAQAAYEYEAQLQANFPYSEELQTVLTGQ----------------- 253
VLNDMLYPTPFIAQ-GNLGWAYYKKGEPDRAVESIKAAVTTNPNFCLGYKNLGLIYDETGRTSEACRQFTHYRENCPDVAEAYMREGVCQAKLGQVDAAKAAFATCETKAKAGEQVLKDDCRRLLEKL------------- 253
......170.......180.......190.......200.......210.......220.......230.......240.......250.......260.......270.......280.......290.......300.
```

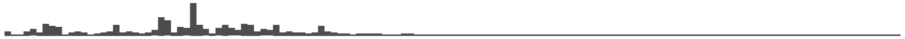

Supplement: Supplementary Figure 2 — Alignment between the aminoacid sequences of TPR proteins using MUSCLE v(3.8.31) (Edgar, 2004). [file Image2.PDF]
